# Sperm epigenetic alterations contribute to inter- and transgenerational effects of paternal exposure to long-term psychological stress via evading offspring embryonic reprogramming

**DOI:** 10.1038/s41421-021-00343-5

**Published:** 2021-10-27

**Authors:** Xiaoguo Zheng, Zhenhua Li, Guishuan Wang, Hanshu Wang, Yuchuan Zhou, Xinzhi Zhao, C. Yan Cheng, Yunbo Qiao, Fei Sun

**Affiliations:** 1 International Peace Maternity & Child Health Hospital, School of Medicine, Shanghai Jiao Tong University, Shanghai, China; 2 Shanghai Key Laboratory of Embryo Original Disease, Shanghai, China; 3 Medical School, Institute of Reproductive Medicine, Nantong University, Nantong, Jiangsu China; 4 The Mary M. Wohlford Laboratory for Male Contraceptive Research, Center for Biomedical Research, Population Council, 1230 York Ave, New York, NY USA; 5 Precise Genome Engineering Center, School of Life Sciences, Guangzhou University, Guangzhou, China; 6 Key Laboratory of Fertility Preservation and Maintenance of Ministry of Education, School of Basic Medicine, Ningxia Medical University, Yinchuan, China

**Keywords:** Epigenetic memory, DNA methylation

## Abstract

Paternal life experiences impact offspring health via germline, and epigenetic inheritance provides a potential mechanism. However, global reprogramming during offspring embryogenesis and gametogenesis represents the largest hurdle to conceptualize it. Yet, detailed characterization of how sperm epigenetic alterations carrying “environmental memory” can evade offspring embryonic reprogramming remains elusive. Here, mice exposed to long-term restraint stress were employed to study the mechanisms underlying inter- and transgenerational effects of paternal exposure to a long-term psychological stress. We found that stress could induce paternal inheritance of reproductive, behavioral, and metabolic disorders. Bisulfite methylation profiling of 18 sperm and 12 embryo samples of three consecutive generations identified inter- and transgenerational inheritance of paternal Differential DNA Methylation Regions (DMRs) at frequencies ~11.36% and 0.48%, respectively. These DMRs related to genes with functional implications for psychological stress response, and tissue inheritance of these DMRs passed paternal disorders epigenetically to offspring. More importantly, these DMRs evaded offspring embryonic reprogramming through erasure and subsequent reestablishment, but not via un-erasure way. Nonetheless, their reestablishment proportions in the primitive streak (E7.5) stage were altered. Furthermore, sncRNA-seq revealed that stress-induced tsRNA, miRNA and rsRNA dysregulation in paternal sperm might play important roles in DMRs occurrence and paternal inheritance. These finding implied that sperm epigenetic alterations contribute to inter- and transgenerational effects of paternal exposure to long-term psychological stress, and highlighted the possible underlying molecular mechanism.

## Introduction

Human epidemiological studies and animal models provide strong evidence supporting the hypothesis that parental life experiences, such as hunger^1,2^, unhealthy diet or nutrient deficiency^3–5^, exposure to toxicants^6–8^, psychological stress^9–11^, and ectopic expression of endogenous genes^12,13^, exert a far-reaching influence on their descendants. Chronic psychological stress, a pervasive problem in society, has been reported to affect metabolism and male fertility in humans, which in turn leads to weight loss, gluconeogenesis disorders, and motivated behavior depression based on studies in rodent models^10,14–18^. Under certain circumstances, psychological effects can be paternally inherited by offspring^10,16^. Such transmission usually does not result from alterations in the primary DNA sequences, and multiple mechanisms have been proposed to account for non-DNA sequence-based inheritance in mammals. These include chemical modifications of DNA and histones, or transfer of small regulatory RNAs complementary to genomic sequences^19^. These widely studied epigenetic marks add another layer of genome information and provide a source of heritable phenotypic changes that is non-DNA primary sequence-based^20^.

Epigenetic inheritance, a germline transmission of epigenetic information from parents to subsequent generations in the absence of both sustained environmental exposures and alterations of genomic DNA sequences, provides a potential mechanism enabling parents to transfer information to their offspring about the environment they experienced^21,22^. The transgenerational inheritance refers to the programming effects being passed across generations in the absence of exposure to the original trigger to either the developing fetus or the germ cells that will eventually become the fetus, i.e. at least three generations for the pregnant maternal linage, but for two generations for the paternal and non-pregnant maternal linages^23–25^. Meanwhile, transmission of the programming effects from F0 to F2 for the pregnant maternal linage and from F0 to F1 for the paternal and non-pregnant maternal linages are considered as the intergenerational inheritance^25^. Epigenetic marks, including DNA methylation, histone modifications, and small non-coding RNAs (sncRNAs), have been found to play critical roles in transgenerational and intergenerational inheritance of environmental or endogenous factors-induced phenotypic alterations in animals^5,10,12^.

DNA methylation, a ubiquitous and conserved epigenetic marker, has recently become a major focus of studies on paternal epigenetic inheritance in mammals. Despite as relatively stable in somatic cells during adult life, the global reprogramming events, which is a key process with DNA methylation patterns reset, occur in early embryos and germ cells during embryogenesis and gametogenesis, respectively^26,27^. Any perturbation in this process likely affects not only current but also future generations^20^. These two waves of epigenetic resetting leave little chance for inheritance of DNA methylation changes, whether accidental or environmentally induced. Therefore, the reprogramming process between generations represents the largest hurdle to conceptualize epigenetic inheritance. In short, epigenetic inheritance has to be reconciled with reprogramming. To date, besides unknown heritable proportions of environmental factors (such as psychological stress) induced sperm DNA methylation changes, it has remained elusive how epigenetic alterations evade global reprogramming, via either erasure and subsequent reestablishment or via un-erasure during the reprogramming process, to mediate paternal inheritance of environmental risk-induced disorders. In addition, many retrotransposons and imprinted genes are also resistant to such global epigenetic reprogramming via hitherto undefined mechanisms^28^. Do heritable DNA methylation changes, which are induced by environmental or endogenous factors, share the same manner as retrotransposons and imprinted genes to get across global reprogramming? These questions require further investigations.

In addition, recent studies have shown that sncRNAs, such as miRNAs (microRNAs), tsRNAs (tRNA-derived small RNAs), and rsRNAs (rRNA-derived small RNAs) can mediate inheritance of environmental-factors-induced phenotypic changes in mammals in a similar manner to that of the more widely studied DNA methylation^5,7,29–32^. These would be due to the ability of sncRNAs to promote activation or repression at transcription sites upon base-complementation pairing with the genetic sequence^10^. It has been reported that sncRNAs, such as piRNAs (piwi RNAs), could mediate establishing DNA methylation of certain targets^33^. DNA methyltransferase 1 (DNMT1) binding of certain miRNAs could induce aberrant DNA methylation of the genome^34^. Mouse mature sperm contained the highest concentration of tsRNAs and displayed the highest tsRNA/miRNA ratio^35,36^. tsRNAs have been shown to participate in mediating intergenerational inheritance of high-fat diet-induced metabolic disorders in mice^5^. Biogenesis of tsRNAs and rsRNAs in sperm are rapidly affected by diet, subsequently mediating changes in sperm motility as well as influencing male reproductive health in humans^37^. Furthermore, alteration of sperm tsRNA and rsRNA expression profile abolished sperm sncRNA-mediated transmission of high-fat-diet-induced metabolic disorders to offspring^38^. As such, subpopulations of sperm sncRNAs could be used as sperm quality biomarkers for in vitro fertilization^39^. Whether psychological stress-induced specific enrichment of sncRNAs in paternal sperm, and whether they are associated with epigenetic inheritance of DNA methylation changes, have yet to be demonstrated.

Most of the earlier studies sought to use a transitory psychological stress treatment (such as for a duration less than a spermatogenic cycle) on adult individuals. As such, offspring would not be produced by the stress-altered germ cells, since there were mature sperm found in experimental mice before treatment, and the stress-altered spermatids might not have matured when these mice were used to produce new offspring in their studies^10,14–17^. Thus, it was difficult to evaluate if it was the stress-induced DNA methylation changes stored in paternal germ cells that mediated the paternal inheritance of the environmental risk factors. Here, a mouse model in which the male mice were subjected to an unprecedented long-term psychological stress from 3 weeks of age was employed to investigate the molecular mechanisms underlying paternal inheritance of psychological post-stress effects across generations.

## Results

### Long-term psychological stress induces paternal inheritance of health risks in mice

**Fig. 1:**
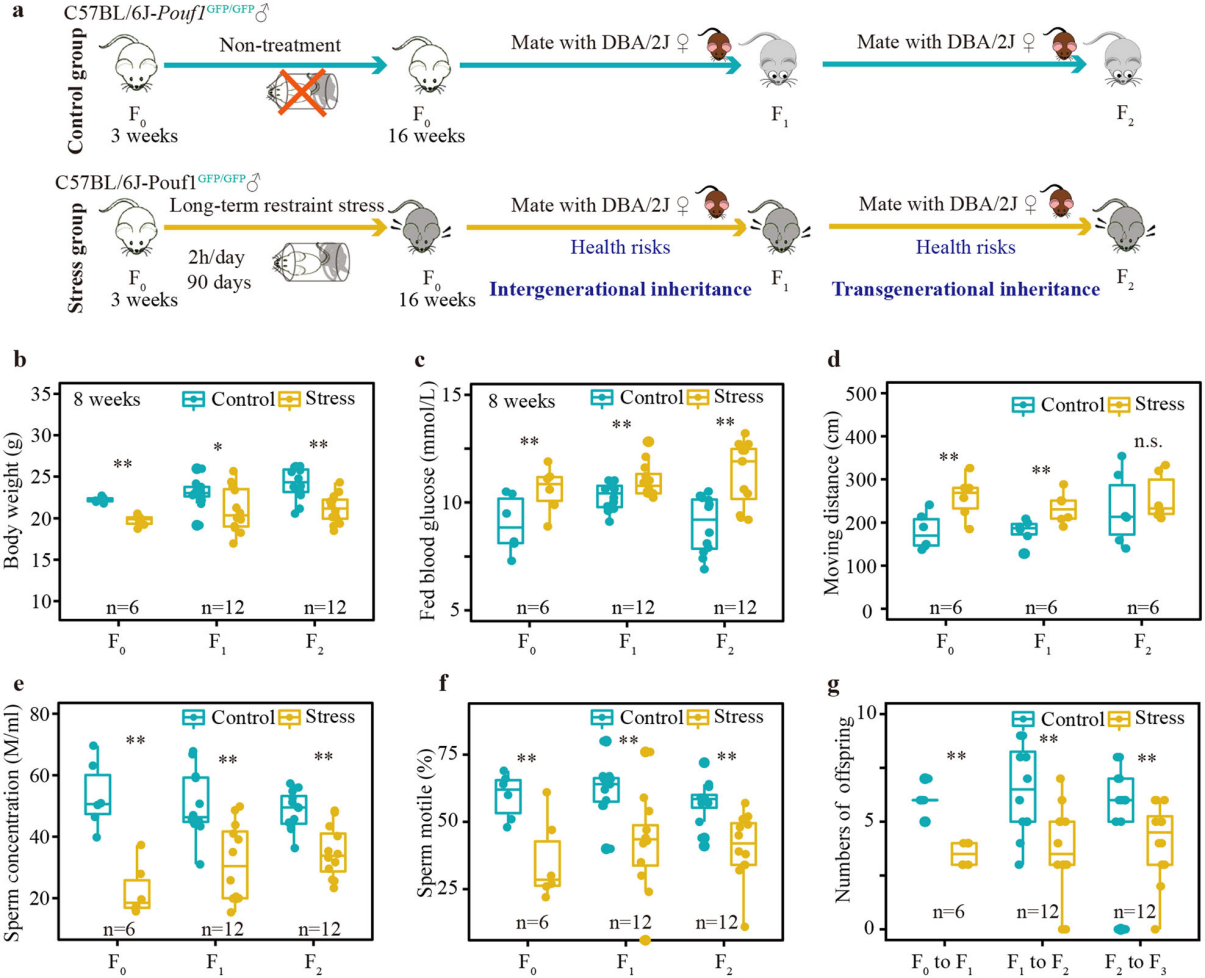
Long-term psychological stress induces paternal inheritance of health risks in offspring. **a** Chronic restraint stress mouse model construction. **b** Stress-induced transgenerational inheritance of developmental retardation. **c** Stress-induced transgenerational inheritance of disorder in blood glucose metabolism. **d** Elevated plus maze (EPM) test to assess anxiety-like behavior in mice. Stress-induced intergenerational inheritance of higher risk-taking behavior and overall physical activity. **e** Stress-induced transgenerational inheritance of decline in sperm concentration decline. **f** Stress-induced transgenerational inheritance of reduced in sperm motility. **g** Stress-induced transgenerational inheritance of reduced in fertility rate. *t-test *p*-value < 0.05; **t-test *p*-value < 0.01; n.s. no significant, *t*-test *p*-value > 0.05.

In mice, it takes ~35 days for spermatogonia stem cells to undergo the differentiation steps to produce spermatozoa, and the first batch of round spermatids begins to appear at 3 weeks after birth^40^. In the present study, we exerted chronic restraint stress on 3-week-old male mice (C57BL/6J*-Pouf1*^GFP/GFP^, the F_0_ generation) for 90 days to ensure that the F_1_ offspring were definitely produced by stress-treated paternal germ cells (Fig. 1a). With respect to our concentration on paternal inheritance, we only examined the phenotypic alteration of the male descendants in subsequent analyses.

Determination of body weights and blood glucose concentrations of all three generations revealed transgenerational inheritance of developmental retardation and increased blood glucose levels in the stressed group, suggesting faithful transmission of stress-induced developmental and metabolic disorders (Fig. 1b, c and Supplementary Fig. S1a, b). Since the restraint stress model has been used for modeling depression and anxiety in animals^15,16^, we also assessed the depression and anxiety-like behaviors in the stress group. Surprisingly, stress-treated mice displayed lower anxiety and were associated with higher risk-taking behavior in both the elevated plus maze test and the open field test, as they spent more time in the central/open region than the control group (Supplementary Fig. S1c, d). Meanwhile, a longer moving distance indicated a higher level of overall physical activity of the stress group (Fig. 1d and Supplementary Fig. S1e). Moreover, these stress-induced behavioral disorders were intergenerationally inherited by the F_1_ generation but vanished in the F_2_ generation (Fig. 1d and Supplementary Fig. S1c–e). Additionally, we investigated sperm quality and reproductive rates of both groups to identify whether long-term stress affected male fertility. The results showed that the sperm concentrations, motile proportions, and numbers of offspring were considerably reduced in the stress group (Fig. 1e–g). Simultaneously, the impaired reproductive ability could be transgenerationally inherited (Fig. 1e–g). Taken together, long-term stress-induced paternal inheritance of health risks in mice, including intergenerationally inherited behavioral disorders and transgenerationally inherited developmental, metabolic, and reproductive disorders, suggesting an epigenetic inheritance of acquired traits.

### Long-term psychological stress stores a lot of “epigenetic memory” in mouse germ cells

To identify stress-induced DNA methylation changes that were stored in paternal germ cells and the portions that were transmitted to descendants, we profiled the sperm DNA methylation patterns of three consecutive generations (F_0_, F_1_, and F_2_) in both control and stress groups by using whole-genome bisulfite sequencing (WGBS). A total of 18 sperm samples were analyzed, including three biological replicates for each generation under each treatment (Supplementary Table S1). On average, we obtained ~800 million clean reads for each sample with strand-specific coverage ~21×, and the data covered ~96.00% of the total 21,867,837 reference CpG dinucleotides (Supplementary Table S1).

**Fig. 2:**
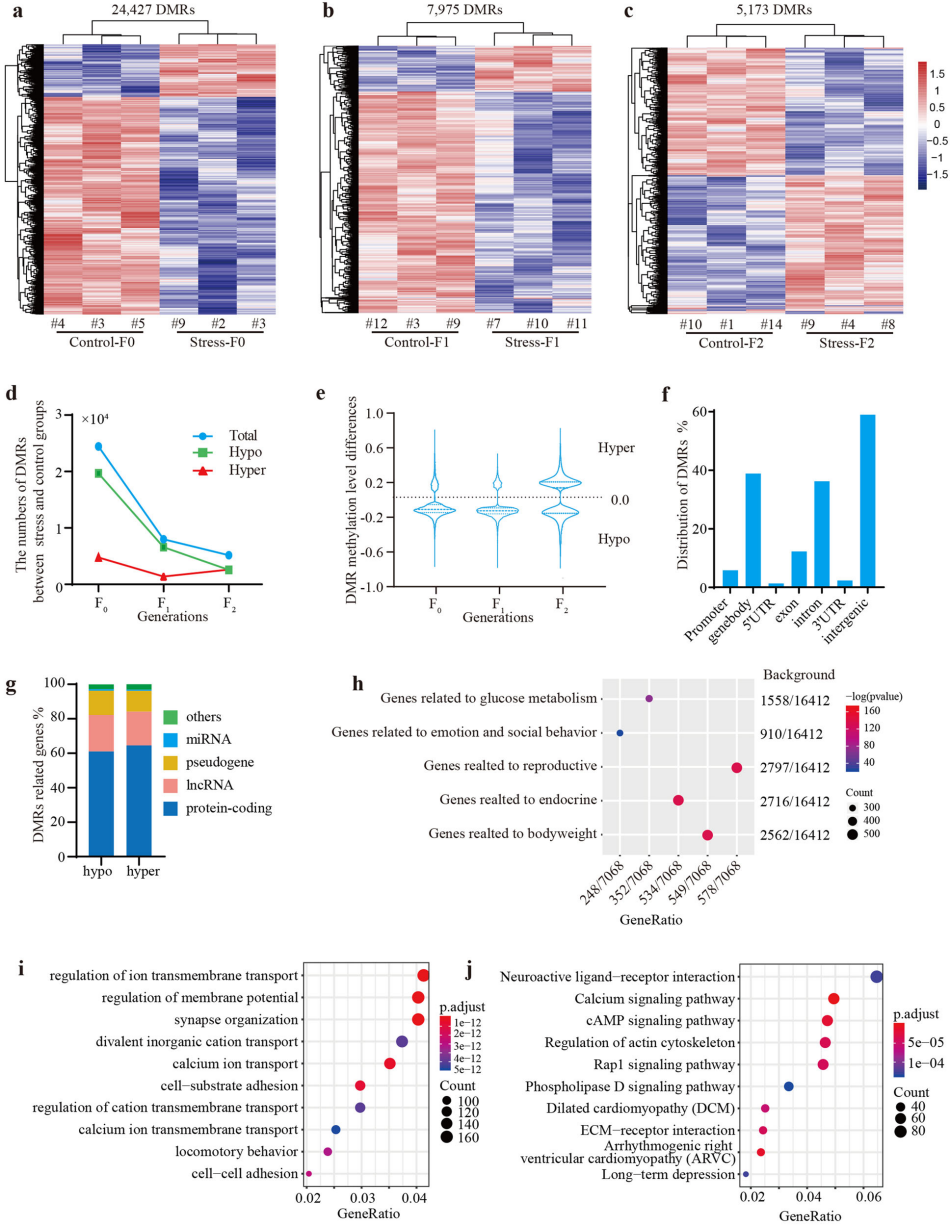
Long-term psychological stress induces storage of “epigenetic memory” in paternal germ cells. **a** Heatmap of the DMRs in the F_0_ generation. **b** Heatmap of the DMRs in the F_1_ generation. **c** Heatmap of the DMRs in the F_2_ generation. **d** Trend in DMRs across generations after removal of the original stimulus – psychological stress. **e** Distribution of the Δβ values (changes in DNA methylation levels of the DMRs) across generations. **f** Distribution of the stress-induced F_0_ DMRs at the gene level. **g** Distribution of the functional genes related to DMRs. **h** Enrichment of F_0_-DMRs related genes to the phenotype-associated genes from mouse genome information (MGI) database. **i** Gene Ontology (GO) analysis of genes related to F_0_-DMRs. **j** Encyclopedia of Genes and Genomes (KEGG) pathway analysis of genes related to F_0_-DMRs.

A total of 24,427, 7975, and 5173 differentially methylated regions (DMRs) between control and stress groups were found in the F_0_, F_1_, and F_2_ generations, respectively (Fig. 2a–c). These data suggested that after the initial environmental stimulus (i.e., psychological stress) was removed, the numbers of DMRs in the descendants dramatically decreased (Fig. 2d). Changes in DNA methylation levels (|Δβ|value) were ~0.1–0.2 in all three generations (Fig. 2e). Meanwhile, the majority of DMRs in both F_0_ and F_1_ generations were de-methylated (Fig. 2d, e).

A significant proportion of DMRs in the F_0_ generation (F_0_-DMRs) were distributed in genes’ promoters and bodies (Fig. 2f and Supplementary Table S2). To investigate whether these DMRs could be associated with stress response, we conducted functional analyses of their related genes, most of which were protein encoding genes (account for ~60%), long non-coding RNAs (lncRNAs, account for ~21%), or pseudogenes (account for ~15%, Fig. 2g and Supplementary Table S2). Enrichment analysis determined that these genes were highly correlated with phenotypic changes, indicating a remarkable association between stress-induced DMRs and stress-induced health risks (Fig. 2h). In addition, Gene Ontology (GO) analysis showed that these genes participated in the regulation of multiple biological processes, including ion transmembrane transport, synapse organization, and locomotory behavior (Fig. 2i). Interestingly, Kyoto Encyclopedia of Genes and Genomes (KEGG) pathway analysis revealed that they were largely involved in the calcium signaling pathway, cAMP signaling pathway, Rap1 signaling pathway, phospholipase D signaling pathway, and long−term depression (Fig. 2j). Furthermore, we performed a protein–protein-interaction (PPI) analysis based on the STRING database to determine whether these genes were functionally clustered and interacted with each other. The results showed that 6664 F_0_-DMRs-related genes were involved in 1170 PPI items with an enrichment *p*-value < 1.0 × 10^−14^, suggesting significant functional enrichment of these genes. Meanwhile, 32 densely connected subnetworks were found based on the topology. These networks were involved in chromatin remodeling (Supplementary Fig. S2a, b), the insulin-like receptor signaling pathway (Supplementary Fig. S2c, d), reflexes and negative regulation of behavior (Supplementary Fig. S2e, f). These functions were also associated with the mouse responses to chronic psychological stress, including changes in global epigenetic modification, abnormities in gluconeogenesis, and behavioral disorders. These results demonstrated that genes related to the DMRs were involved in a wide range of functions and markedly correlated with stress-induced health risks. Therefore, long-term restraint stress, representing psychosocial experience during the paternal lifespan, could not only induce many health risks but could also store a great number of DMRs that represented the “epigenetic memory” in the germ cells.

### A notable proportion of “epigenetic memory” is paternally inherited across generations

**Fig. 3:**
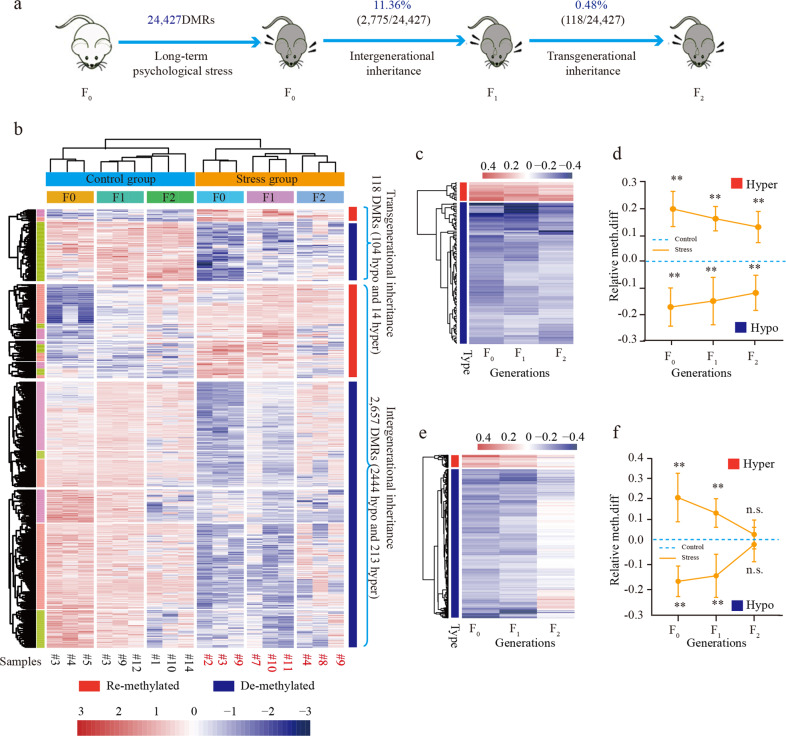
Epigenetic inheritance of “epigenetic memory” across generations. **a** Proportions of the intergenerationally and transgenerationally inherited DMRs across generations. **b** Heatmap of the DNA methylation status of all epigenetically inherited F_0_-DMRs. **c** Heatmap of the Δβ values of the transgenerationally inherited DMRs. **d** Tendencies of the Δβ values of the transgenerationally inherited DMRs in advanced generations. **e** Heatmap of the Δβ values of the intergenerationally inherited DMRs. **f** Tendencies of the Δβ values of the intergenerationally inherited DMRs in advanced generations. **t-test *p*-value < 0.01; n.s. no significant, t-test *p*-value > 0.05.

We speculated that paternal inheritance of stress-induced health risks would be mediated by epigenetic inheritance of the “epigenetic memory” that was stored in paternal sperm. To verify this conjecture, we analyzed the DNA methylation status of the F_0_-DMRs in all samples of both F_1_ and F_2_ generations (Supplementary Table S2). On the basis of DNA methylation levels of each generation (three individual replicates), 11.36% F_0_-DMRs (2,775/24,427; *P* < 0.05, unpaired *t*-test; FDR < 0.01) were intergenerationally inherited (i.e., differentially methylated in both F_0_ and F_1_ but not in the F_2_), of which 0.48% (118/24,427) were transgenerationally inherited (i.e., differentially methylated in each of the F_0_, F_1_, and F_2_ generations) (Fig. 3a, b and Supplementary Table S2). This approach seemed to be more accurate than that based on mean DNA methylation levels of all three generations, as the differences in each of the three generations were calculated separately. Although these DMRs were paternally inherited, the differences in their DNA methylation levels between control and stress groups were decreasing in the descendants, suggesting a limited inheritance cycle of these DMRs after the original trigger was removed (Fig. 3c–f).

### Tissue-specific expression of genes related to sperm-inherited DMRs correlate with paternal inheritance of health risks

**Fig. 4:**
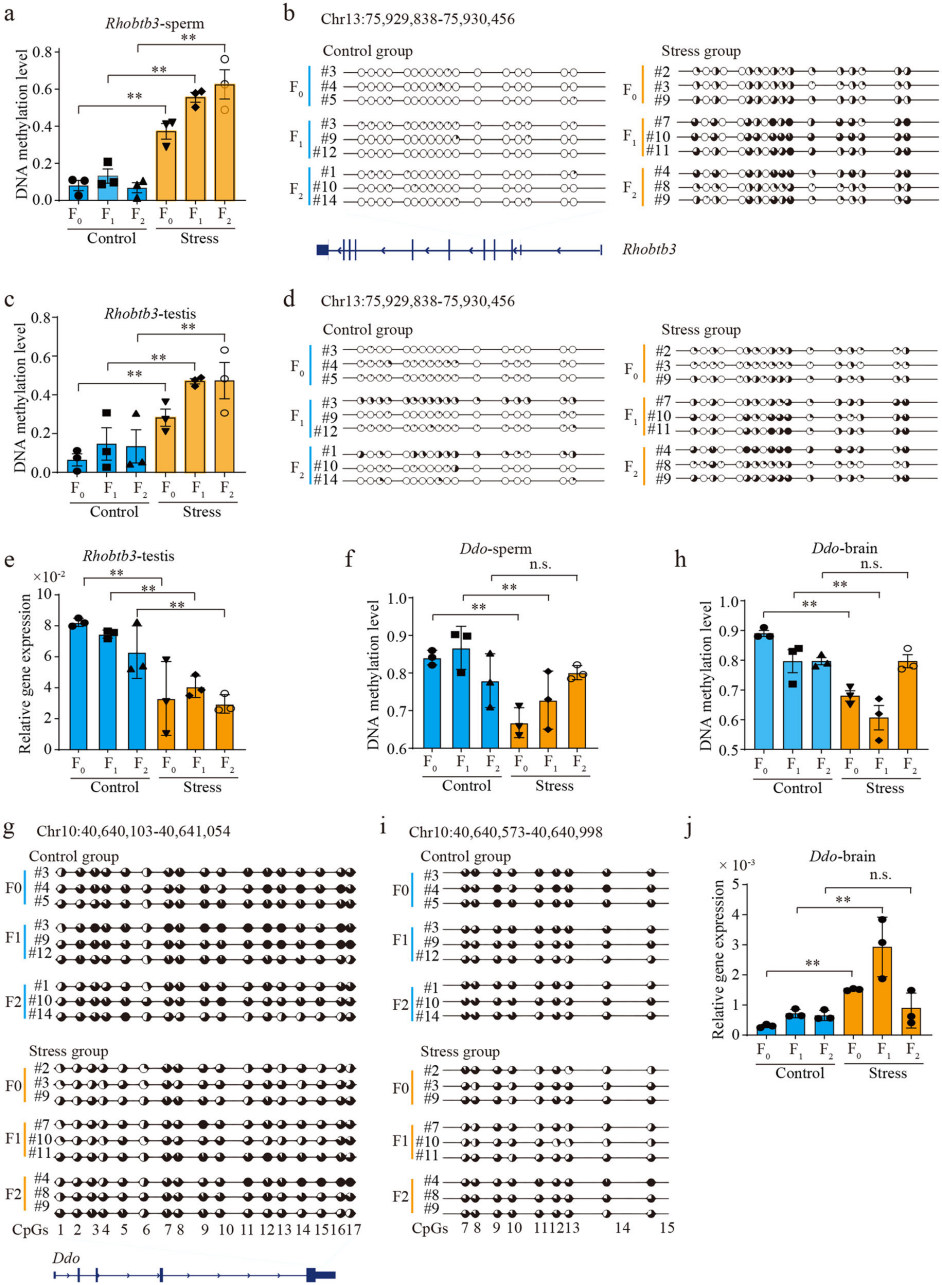
Epigenetically inherited DMRs correlate with paternally inherited health risks. **a** DNA methylation levels of the transgenerationally inherited DMR, Chr13:75,929,838-75,930,456, in sperm samples of all generations. **b** DNA methylation patterns of the transgenerationally inherited DMR in sperm samples. **c** DNA methylation levels of the transgenerationally inherited DMR in testis tissues. **d** DNA methylation patterns of the transgenerationally inherited DMR in testis tissues. **e** Gene expression patterns of *Rhobtb3* in testis tissues. **f** DNA methylation levels of the intergenerationally inherited DMR, Chr10:40,640,103-40,641,054, in sperm samples of all generations. **g** DNA methylation patterns of the intergenerationally inherited DMR in sperm samples. **h** DNA methylation levels of the intergenerationally inherited DMR in brain tissues. **i** DNA methylation patterns of the intergenerationally inherited DMR in brain tissues. **j** Gene expression patterns of *Ddo* in brain tissues. **t-test *p*-value < 0.01; n.s. no significant, *t*-test *p*-value > 0.05.

To investigate whether these epigenetically inherited DMRs correlated with the paternal inheritance of stress-induced health risks, we detected their DNA methylation status as well as expression patterns of their related genes in tissues. *Rhobtb3* was previously found as the most divergent member of the RhoBTB subfamily, atypical Rho GTPases within the Rho family, and is expressed in spermatocytes and spermatids in the testis^41,42^. Its deficiency was associated with reproductive disorders in mice. Here, it was related to a transgenerationally inherited re-methylated DMR (Chr13:75,929,838–75,930,456, located at exon, Fig. 4a, b). Bisulfite sequencing PCR (BSP, see Methods section) revealed that changes in DNA methylation levels and patterns of *Rhobtb3*-related DMR was transmitted through the germline but also through the testis tissue (Fig. 4c, d). Moreover, its expression level was considerably reduced, and this pattern was transgenerationally inherited by offspring and was closely related to stress-induced developmental and reproductive disorders (Fig. 4e). Another gene, *Il12rb1*, was known to be associated with mouse adipose metabolism and development. Its homologous gene, *Il13ra2*, has been reported to mediate paternal inheritance of chronic high-fat diet-induced β-cell dysfunction in female offspring in rats^43^. This gene was also found to be related to a transgenerationally inherited de-methylated DMR (Chr8:70,819,260-70,819,910, located at the splice region of intron, Supplementary Fig. S3a, b and Supplementary Table S2). Similar to the *Rhobtb3*-related DMR, its changes in DNA methylation levels and patterns were also noted in liver tissue (Supplementary Fig. S3c, d). Furthermore, the up-regulation pattern in the liver was transgenerationally inherited by descendant tissues and was associated with metabolic disorders (Supplementary Fig. S3e).

Additionally, two protein-coding genes, *Ddo* and *Oprm1*, were also found to be related to two intergenerationally inherited de-methylated germline DMRs, Chr10:40,640,103-40,641,054 (located at 3′UTR) and Chr10:6,886,538-6,887,843 (located at 3′UTR), respectively (Fig. 4f, g, Supplementary Fig. S3f, g). A high expression level of *Ddo* has been reported in the mouse brain, and *Ddo*^−/−^ mice displayed deficits in behavior^44^. *Oprm1* has been reported to mediate anxiety-related behavior and social approach in a mouse model of MECP2 duplication syndrome, and reduced *Oprm1* expression improved abnormal social behavior^45^. We found that the DNA methylation patterns and the levels of these DMRs could be intergenerationally inherited by F_1_ tissues (Fig. 4h, i and Supplementary Fig. S3h, i). More importantly, the related genes were up-regulated in both the F_0_ and F_1_ brains, and the levels strongly correlated with stress-induced behavioral disorders (Fig. 4j and Supplementary Fig. S3j). These findings suggested that epigenetically transmitted germline DMRs, including intergenerationally and transgenerationally inherited forms, could also be transmitted to relevant tissues across generations. Furthermore, these tissue-inherited DMRs were possibly responsible for paternal inheritance of health risks through altering expression patterns of their related genes in relevant tissues.

### Heritable DMRs are erased and subsequently reestablished, but not unaltered, to get through offspring embryonic reprogramming

**Fig. 5:**
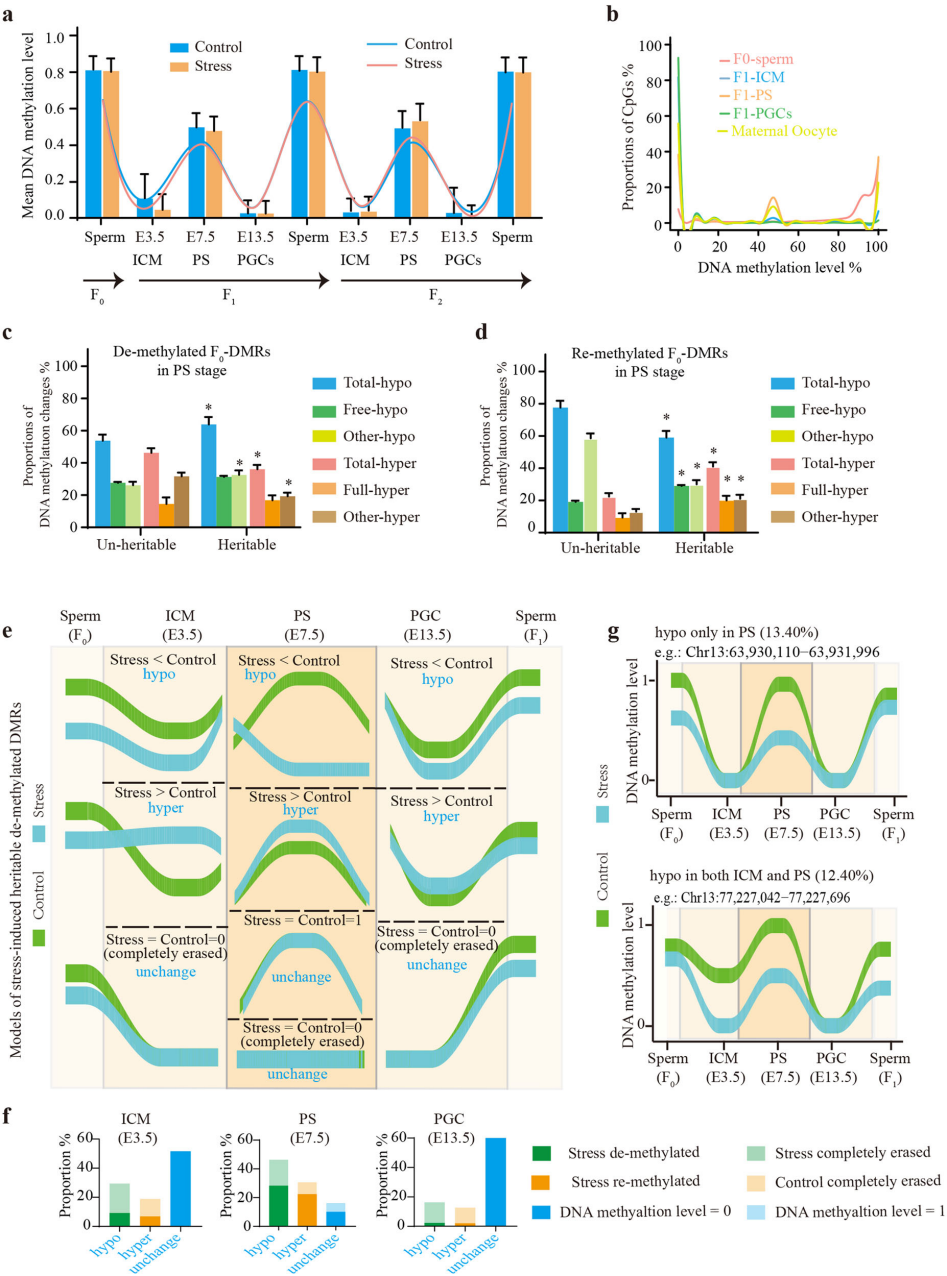
Heritable DMRs have alterative DNA methylation reestablishment proportions and levels in the PS stage during embryonic reprogramming process. **a** Mean DNA methylation levels in different embryo stages and sperm samples. **b** Distribution of the CpG dinucleotides in different DNA methylation levels. **c** Alterations in DNA methylation patterns of both the heritable and the un-heritable de-methylated F_0_-DMRs when compared with that of the paternal sperm samples during reprogramming process of the F_1_ generation. Total de-methylated (Total-hypo) events were composed of free-methylated (Free-hypo) events and the other de-methylated (Other-hypo) events, while total re-methylated (Total-hyper) events were composed of full-methylated (Full-hyper) events and the other re-methylated (Other-hyper) events. **d** Alterations in DNA methylation patterns of both the heritable and the un-heritable re-methylated F_0_-DMRs when compared with that of the paternal sperm samples during the reprogramming process of the F_1_ generation. **e** Dynamic DNA methylation patterns of the stress-induced heritable de-methylated DMRs during reprogramming process. In each stage, all DMRs were classified into three types: hypo (stress group was de-methylated), hyper (stress group was re-methylated), and unchanged (there was no difference between two groups). Theoretically, there were 36 combinations of the reprogramming patterns of these DMRs. **f** Proportion of each DMR type in each embryo stage. **g** The top two reprogramming patterns of the DMRs that were sequencing covered in all embryo samples of both the control and stress groups.

Although a notable proportion of stress-induced DMRs were intergenerationally or transgenerationally transmitted, the underlying mechanisms by which they got through global reprogramming during embryogenesis and gametogenesis remained elusive. Here, we performed single-cell WGBS (scWGBS) for 12 embryo samples, including ICM (Inner cell mass, E3.5), PS (Primitive streak. E7.5), and PGCs (Primordial germ cells, E13.5) of both F_1_ and F_2_ generations in the two groups, and for one oocyte sample from the maternal strain (Supplementary Table S1). These data illustrated two waves of DNA methylation resetting (erasure and then reestablishment) occurred during embryogenesis (from zygote to the offspring PS stage) and gametogenesis (from the PS stage to the offspring mature sperm) (Fig. 5a), consistent with findings of a recent report^26^. Importantly, profiling of DNA methylation patterns in all samples revealed that most of the CpG dinucleotides in parental germ cells (including both sperm and oocytes) were highly methylated (methylation level >80%), while most were unmethylated in the ICM and PGCs (Fig. 5b). In the PS, we observed that low-methylated and high-methylated CpG dinucleotides were present in similar proportions (Fig. 5b).

Two waves of DNA methylation reprogramming implied that the vast majority of paternal lifespan experiences were not transmitted to offspring through attached-epigenetic information in the germ cells. To investigate the dynamic DNA methylation status of the heritable DMRs during reprogramming, we merged the information of all the DMRs transmitted from the F_0_ generation to the F_1_ generation, or from the F_1_ generation to the F_2_ generation, to analyze the patterns of intergenerational and transgenerational inheritance, respectively. Meanwhile, an equal-sized set of the F_0_-DMRs that were not inherited by descendants (un-heritable F_0_-DMRs) was treated as the control group. The dynamic DNA methylation status of all embryonic samples were compared with that of the paternal sperm, which was regarded as possessing the original methylation pattern. The DMRs were then classified into seven categories, including “total-hypo” (representing de-methylated DMRs in embryo samples in comparative analysis with the paternal sperm), “free-hypo” (representing unmethylated DMRs which DNA methylation status were completely erased in embryo samples among “total-hypo” DMRs), “other-hypo” (representing the other de-methylated DMRs besides “free-hypo”), “total-hyper” (representing re-methylated DMRs in embryo samples in comparative analysis with the paternal sperm), “full-hyper” (representing full-methylated DMRs among “total-hyper” DMRs, which were completely methylated in embryo samples), “other-hyper” (representing the other re-methylated DMRs besides “full-hyper”), “unchanged” (representing DMRs that the DNA methylation status were not changed in embryo samples in comparative analysis with the paternal sperm). We observed more occurrence of the “total-hypo” DMRs, including the “free-hypo” and the “other-hypo” DMRs, than the “total-hypo” DMRs, including the “full-hyper” and the “other-hyper” DMRs, in all three embryo stages of both control and stressed groups (Supplementary Fig. S4a, b). In addition, a higher proportion of “free-hypo” events and a lower proportion of “total-hyper” events were noted in the PGCs than those in the ICMs, indicating more thoroughly demethylation in the former. In the PS, the “total-hypo” DMRs were evenly composed of “free-hypo” DMRs and “other-hypo” DMRs, while the “total-hyper” DMRs were dominated by the “full-hyper” DMRs (Supplementary Fig. S4a, b).

There were no significant differences in DNA methylation patterns between the heritable and un-heritable F_0_-DMRs in both the ICM and PGC stages in comparative analysis with the paternal sperm using Fisher’s exact test (Supplementary Fig. S4c–f). However, in the PS stage, the heritable de-methylated F_0_-DMRs had significantly more “total-hypo” events, whereas there were fewer “total-hyper” events than in the un-heritable de-methylated F_0_-DMRs in both the F_1_ and F_2_ generations (Fig. 5c and Supplementary Fig. S4g). In contrast, the heritable re-methylated F_0_-DMRs had markedly fewer “total-hypo” events, while there were more “total-hyper” events than in the un-heritable re-methylated F_0_-DMRs in the PS stage of both the F_1_ and F_2_ generations (Fig. 5d and Supplementary Fig. S4h). These results indicated that heritable DMRs were erased and subsequently reestablished, but not unaltered to get through offspring embryonic reprogramming. However, they had a different reestablishment proportion with the un-heritable DMRs in the PS stage.

Furthermore, we analyzed changes in DNA methylation levels between stress-treated and control groups of these heritable DMRs in all three embryo stages (Fig. 5e and Supplementary Fig. S4i). For heritable de-methylated DMRs, there were more “total-hypo” events than “total-hyper” events in all three embryo stages, especially in the PS stage, indicating that most had a lower reestablished methylation level in the stress-treated group than in the control group (Fig. 5f). Among heritable de-methylated DMRs that were sequencing covered in all F_1_ embryo samples, the reprogramming pattern in which demethylation events only occurred in the PS stage accounted for the largest proportion, while the pattern of demethylation events occurring in both the ICM and PS stages was the second most common (Fig. 5g). In contrast, for heritable re-methylated DMRs, most had a higher reestablished methylation level in the stress group than in the control group in all stages (Supplementary Fig. S4j). The same results were noted in the F_2_ generation (Supplementary Fig. S4k, l). These observations suggested that the heritable DMRs had a different reestablished methylation level in the stress group compared with that in the control group during reprogramming, especially in the PS stage. Taken collectively, these findings suggest that during offspring embryonic reprogramming, heritable DMRs were erased and subsequently reestablished. However, their reestablishment proportions and reestablished methylation levels were altered when compared with un-heritable DMRs and the untreated group.

### Imprinted genes and some transposable elements escape the first round of demethylation

The DNA methylation patterns of the imprinted genes and the transposable elements are known to be stably inherited by subsequent generations. A previous study revealed that the imprinted genes and some repeated elements (e.g., retrotransposons) did not undergo reprogramming during the first round of demethylation^46^. We sought to identify the dynamic DNA methylation status of imprinted genes and transposable elements during offspring embryonic reprogramming. A total of 50 paternally expressed genes (maternally imprinted) and 62 maternally expressed genes (paternally imprinted) were collected from the Imprinted Gene Database (Methods section). Although the DNA methylation levels of the maternal imprinted genes were higher than those in paternal imprinted genes in the sperm of all generations, all showed low methylation levels in sperm but high methylation levels in PS (Supplementary Fig. S5a–c). In contrast to canonical genes, the imprinted genes had less changes in DNA methylation levels between the paternal sperm and offspring ICMs (Supplementary Fig. S5b, c), which may be due to their substantial roles in regulating parent-of-origin gene expression during embryogenesis^26^. However, all of these imprinted genes underwent a more thorough erasure in the PGCs than in the ICM as well as canonical genes (Supplementary Fig. S5b, c).

In addition, we analyzed the dynamic DNA methylation status of transposable elements (TE), including retrotransposons and DNA transposons that were collected from the Dfam database (Materials and methods). Finally, a total of 129,146 long-terminal repeated (LTR) retrotransposons, 132,515 long interspersed nuclear element (LINE) retrotransposons, 129,322 short interspersed nuclear element (SINE) retrotransposons, 13,350 satellite repeats, 2527 pseudogenes, and 60,860 DNA transposons were examined. The results showed that the transposons were highly methylated in all sperm samples, and their reprogramming processes during embryogenesis and spermatogenesis were similar to those of the canonical genes but not to those of the imprinted genes (Supplementary Fig. S5d, e). Interestingly, there were 2–5% transposable elements that escaped demethylation, i.e., keeping their methylation status, in the ICM stage (Supplementary Fig. S5g).

Agouti viable yellow (*A*^*vy*^) and Axin Fused (*Axin*^*Fu*^) loci are two of the best-characterized paradigms of non-genetic inheritance in mammals^47^. In the naturally occurring mutant mice, genetically identical individuals exhibit quantifiable phenotypic variability in coat color or tail morphology due to the insertion of an endogenous retrovirus (ERV) of the intracisternal A particle (IAP) class into the *Agouti* or the *Fused* loci, respectively^48,49^. For *A*^*vy*^ locus, in a C57BL/6 J genetic background, the phenotype of the dam, but not the sire, influences the phenotypic distribution observed in the offspring^47,48^. For *Axin*^*Fu*^ locus, its epigenetic state can be inherited transgenerationally after both maternal and paternal transmission. This is in contrast to epigenetic inheritance at the *A*^*vy*^ allele, which occurs in females only^50^. Although the mouse strains that we used in this study (C57BL/6J-*Pouf1*^*GFP/GFP*^ and DBA/2J) were without insertions of IAPs into the *A*^*xy*^ locus and the *Axin*^*Fu*^ locus, as two classic cases of epigenetic inheritance, we performed additional analysis for these two loci (*A*^*xy*^ locus, chr2:154,951,219-155,051,011; *Axin*^*Fu*^ locus, chr17:26,138,688-26,195,811) based on our datasets. We separately identified the DNA methylation status of their promoter regions (~2 kb upstream from TSS site), exons and introns in 18 sperm samples and 12 embryo samples to investigate their inheritance patterns. The results showed that both *A*^*xy*^ and the *Axin*^*Fu*^ loci were highly methylated in all 18 sperm samples, and no significant inter-individual variation was noted (Supplementary Fig. S5f, g), indicating that the DNA methylation patterns of these two loci were not affected by psychological stress. In order to investigate their inheritance patterns, the sequencing data derived from the same generation (F0, F1, or F2) or from the same embryonic stage (E3.5, E7.5, or E13.5) were merged, respectively. The average DNA methylation level of each CpG site in these two loci of three generations was illustrated in Supplementary Fig. S5h, i, demonstrating that most of them were highly methylated. Meanwhile, their DNA methylation status in three embryonic stages (E3.5, E7.5, and E13.5) were compared with that in paternal sperms, showing that the DNA methylation patterns of these two loci were erased and subsequently reestablished during reprogramming, similar to other canonical genes (Supplementary Fig. S5j, k). In brief, these findings suggest that *A*^*vy*^ and *Axin*^*Fu*^ loci do not escape epigenetic reprogramming upon restraint stress exposure.

In addition, Ann Ferguson-Smith group identifies 105 variably methylated IAPs (VM-IAPs) with *A*^*vy*^ epigenetic properties by using a systematic genome-wide screen method in C57BL/6J murine^47^. The same genetic background (C57BL/6J) allowed us to investigate the inheritance patterns of these VM-IAPs in our datasets. Among these 105 VM-IAPs, there were 18 VM-IAPs with SD (sample standard deviation) >0.1, i.e., these VM-IAPs vary greatly among sperm samples of biological replicates or treatments (Supplementary Fig. S5l and Table S2). Thus, we removed these 18 VM-IAPs for further analysis. Within the remaining 87 VM-IAPs, there were 7455 CpG sites; among these, 6184 sites were covered by sequencing data in sperm samples of all three generations, and most of these CpG sites were highly methylated (DNA methylation level >0.8, Supplementary Fig. S5m). Comparison of the DNA methylation levels of these CpG sites in embryo samples and paternal sperm samples revealed that 88.48% of these CpG sites were de-methylated in ICM (E3.5), among which, 84.85% of them were completely erased (Supplementary Fig. S5n). Subsequently, ~80% of these sites were reestablished in the primitive streak (E7.5), although ~48% of these reestablished sites showed a lower DNA methylation level than that in paternal sperm (Supplementary Fig. S5n). In the PGCs (E13.5), ~90% of these CpG sites were de-methylated and ~80% were completely erased (free-methylated). These results suggested that the DNA methylation patterns of most of the CpG sites in VM-IAPs were also erased and subsequently reestablished during reprogramming, and ~12% of CpG sites escaped from the first wave of demethylation during reprogramming (Supplementary Fig. S5n).

In summary, results reported here suggested that the ICM stage would be the crucial stage for determining epigenetic inheritance of the imprinted genes. Meanwhile, imprinted genes might have different mechanisms of reprogramming with transposon elements (including VM-IAPs) and psychological stress-induced heritable DMRs.

### Small non-coding RNAs are correlated with occurrence and paternal inheritance of stress-induced DMRs

**Fig. 6:**
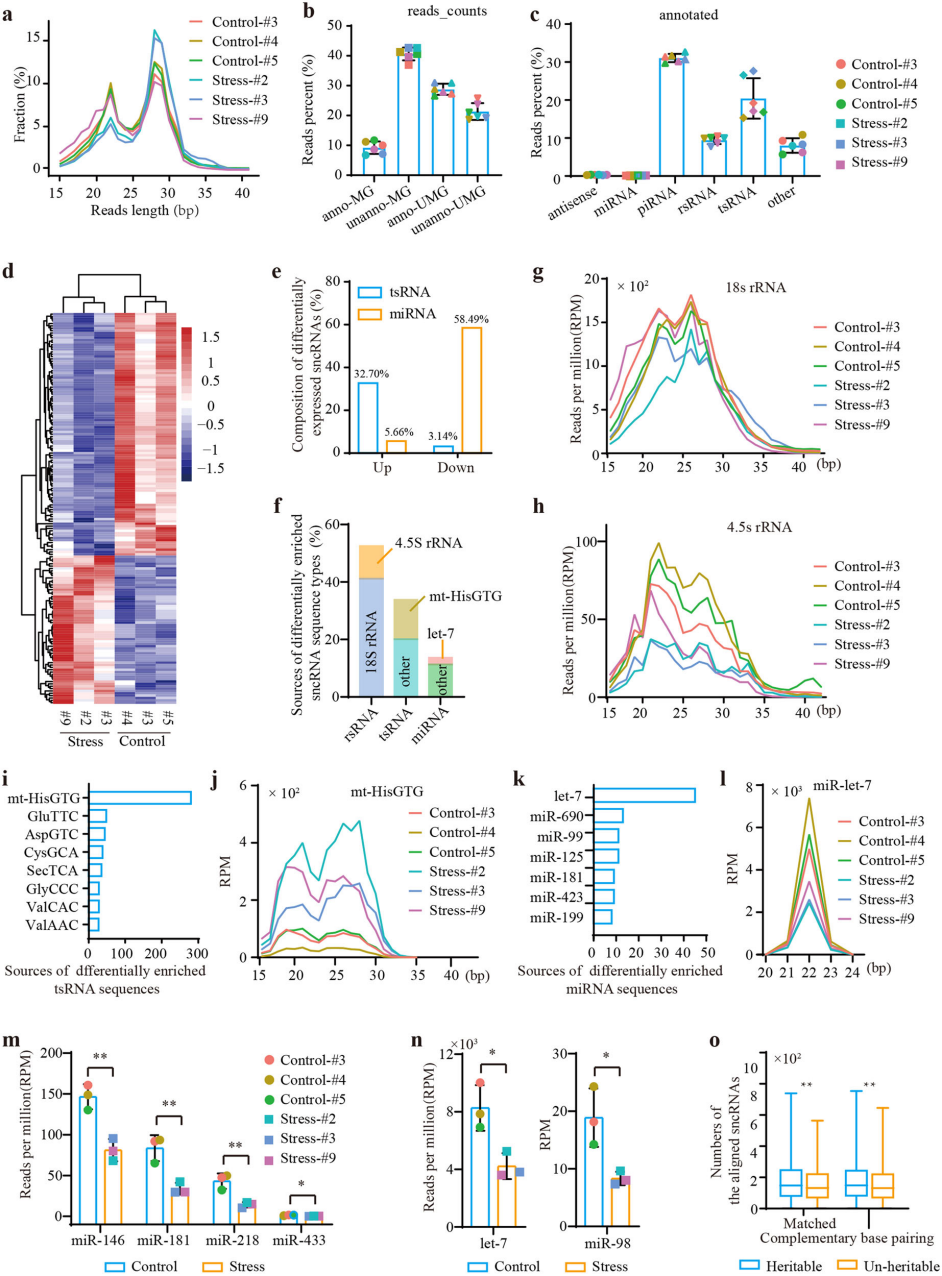
Differentially enriched sncRNAs in paternal sperm. **a** Length distribution of all sequencing reads. **b** Alignment of the sequencing data. anno_MG: matched to the reference genome and annotated; unanno-MG: matched to the reference genome but not annotated; anno-UMG: unmatched to the reference genome but annotated; unanno-UMG: unmatched to the reference genome and not annotated. **c** Distribution of the reads in annotated RNAs. **d** Heatmap of differentially enriched sncRNA subclasses. **e** Composition of the differentially enriched sncRNA subclasses. **f** Sources of differentially enriched sncRNA sequence types. **g** Length distribution of the 18S rRNA-derived rsRNA sequences in paternal sperm. **h** Length distribution of the 4.5S rRNA-derived rsRNA sequences in paternal sperm. **i** The main sources of the differentially enriched tsRNA sequences. **j** Length distribution of the mt (mitochondrial)-HisGTG-derived sequences. **k** The main sources of the differentially enriched miRNA sequences. **l** Length distribution of the miR-let-7-derived sequences. **m** Expression levels of the miRNAs related to *Rhobtb3* gene. **n** Expression levels of the miRNAs related to *Oprm1* gene. **o** Differences in the numbers of the aligned sncRNA sequences between heritable and un-heritable DMRs.

Although the reestablishment patterns of these heritable DMRs were altered in the PS stage, their changed methylation status were almost fully cleared in the ICM and PGCs. Thus, it is likely that there are some other mechanisms “marking” the heritable status of these DMRs. Here, small RNA sequencing was carried out on paternal sperm samples to investigate whether long-term psychological stress affected the enrichment of certain sncRNAs and to identify whether they participated in mediating the occurrence and paternal inheritance of the stress-induced DMRs (Fig. 6a and Supplementary Table S1). The sequencing data were then aligned to a reference genome, a miRNA database, a ribosomal RNA (rRNA) database, a transfer RNA (tRNA) database, a piRNA database, a non-coding (ncRNA) database, and the Rfam database to validate the source of the sncRNAs (Methods section). We found that on average, ~50% of sequencing reads were matched to the reference genome, of which piRNAs and tsRNAs were the most abundant species (Fig. 6b, c). After normalization by reads per million (RPM), 159 differentially expressed subclasses of sncRNAs between the control group and the stress group were enriched, including 98 down-regulated and 61 up-regulated subclasses (Fig. 6d and Supplementary Table S3). Interestingly, the vast majority of the up-regulated sncRNAs subclasses were tsRNAs (52/61, 85.25%), while most of the down-regulated sncRNAs were miRNAs (93/98, 94.90%) (Fig. 6e and Supplementary Table S3). In addition, 4.5S rRNA- and 18S rRNA-derived small RNAs (rsRNAs) were also significantly down-regulated in paternal sperm after stress treatment (Supplementary Table S3). In fact, more than half of the differentially enriched sncRNA sequence types were rsRNAs (Fig. 6f and Supplementary Table S4). Screening according to that sum of the reads count >15 and sum of the RPM value >1 for all 6 samples, and *t*-test *p*-value < 0.05 between the control group and the stress group, we obtained 1017, 655, and 264 differentially enriched sequences related to rsRNA, tsRNA, and miRNA, respectively (Fig. 6f and Supplementary Table S4). Among differentially enriched rsRNAs, 18S-rsRNA, and 4.5S-rsRNA accounted for 78.37% and 21.63%, respectively (Fig. 6f-h). Among the differentially enriched tsRNA sequences, mt-HisGTG, GluTTC, and AspGTC, were the top three isodecoders that were significantly affected by psychological stress (Fig. 6f, i, j and Supplementary Table S4). Among the differentially enriched miRNA sequences, let-7 had the most differentially enriched fragments (Fig. 6f, k, l and Supplementary Table S4). Such differential response patterns of the rsRNA, tsRNA, and miRNA could imply different roles they might have played in reaction to psychological stress.

We then analyzed whether these differentially enriched sncRNAs might have any roles in occurrence and paternal inheritance of stress-induced DMRs. Target prediction based on the miRNA database (TargetScan) revealed that 71.05% and 65.98% of the protein-coding genes related to intergenerationally and transgenerationally inherited DMRs, respectively, were targeted by differentially enriched miRNAs (Supplementary Table S2). For instance, *Rhobtb3* was associated with miR-146, miR-181, miR-218, and miR-433, all of which were remarkably down-regulated in mouse sperm in the stress group (Fig. 6m). *Oprm1* was targeted by two significantly down-regulated miRNAs, let-7 and miR-98-5p (Fig. 6n). Furthermore, we retrieved all sequencing reads related to the differentially enriched sncRNAs, including rsRNAs, tsRNAs, and miRNAs, and performed target prediction for stress-induced DMRs (Materials and methods section). Target prediction revealed that almost all of the F_0_-DMRs were matched with or base-complementation paired by differentially expressed sequences with 8–10 bp regions. Furthermore, the heritable F_0_-DMRs had significantly more partially aligned sncRNAs than the un-heritable DMRs (Fig. 6o). All of these observations suggested that long-term psychological stress-induced enrichment of specific sncRNAs in paternal sperm, including significantly up-regulated tsRNAs and down-regulated miRNAs and rsRNAs. Moreover, sequences related to these differentially expressed sncRNAs possibly participated in mediating the occurrence and paternal inheritance of the stress-induced DMRs.

## Discussion

Psychological stress is one of the most important health and social problems confronting virtually every living individual today. Here, using a mouse model, we found that long-term psychological stress-induced developmental, behavioral, and metabolic disorders in male individuals, consistent with previous studies in humans and rodents^10,14–18^. More importantly, psychological stress has long been suspected of having an important impact on fertility and infertility, but conclusive evidence remains lacking^18^. In the present study, we demonstrated that stress was able to induce paternally inherited reproductive disorders across generations, including diminished sperm quality and a lower fertility rate. Our findings are somewhat different from an earlier study^10^. This could be due to the long-term treatment (more than two cycles of spermatogenesis) in our study, ensuring that the paternal germ cells that produced the F_1_ generation were affected by the psychological stress, different from previous studies in which the model animals were exposed to a transient stress^9,10,14,16,17^. Despite the limitation that the extent and severity of the effects of psychological stress on human tissues is difficult to study, stress as a causative factor in male metabolism, emotional response, and infertility cannot be ignored, and humans should be made aware of its effects on health.

**Fig. 7:**
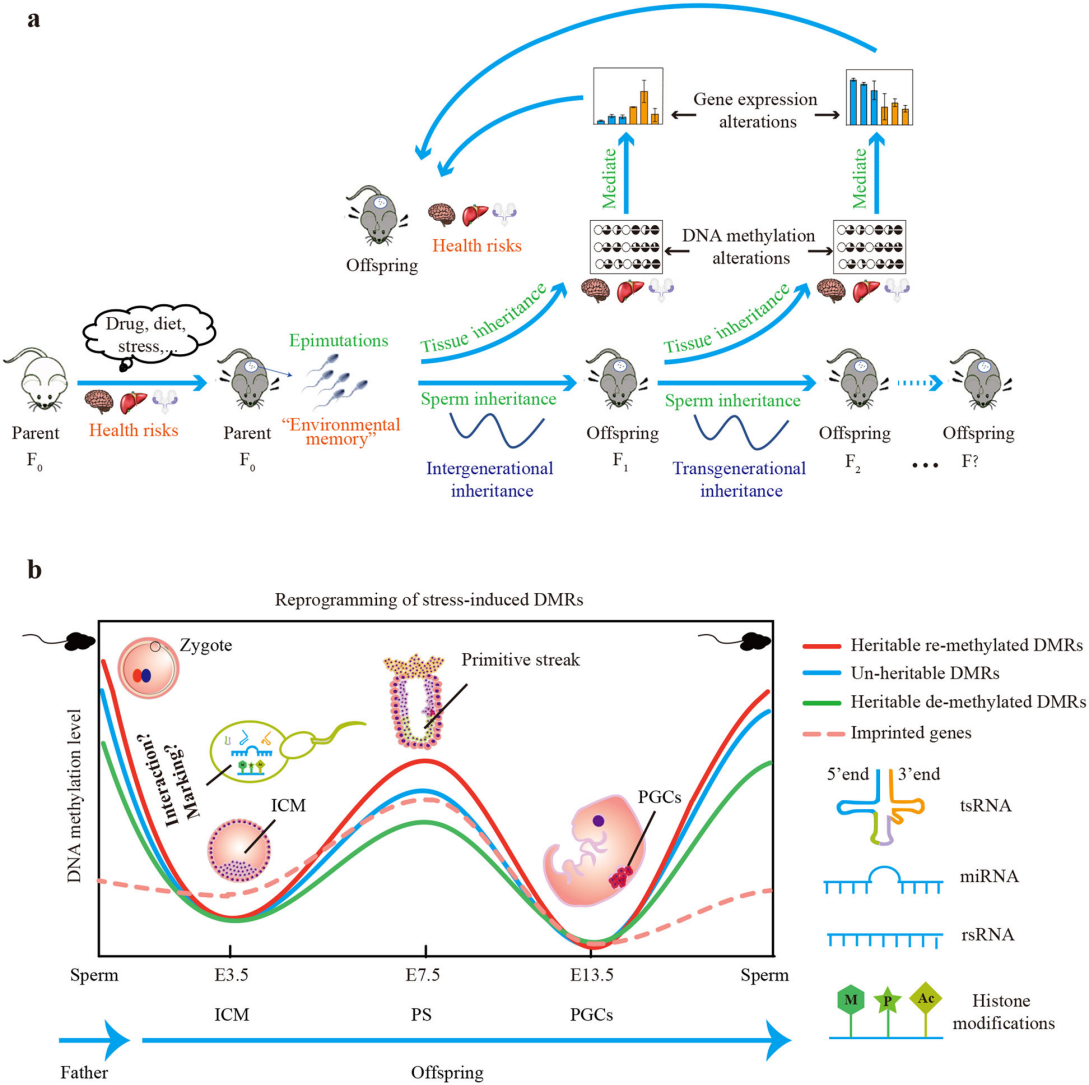
A model illustrating paternal inheritance of psychological post-stress effects. **a** Environmental stimuli, such as long-term psychological stress, could induce health risks in male mice. Simultaneously, a lot of epimutations such as DMRs which represented “epigenetic memory of paternal life experiences” were stored in paternal germ cells. More importantly, notable proportions of these epimutations were epigenetically (including intergenerationally and transgenerationally) inherited by tissues as well as germ cells of the offspring. Subsequently, tissue-inherited epimutations modulated expression patterns of their related genes in relevant tissues, which in turn caused transgenerational transmission of health risks. **b** Most of the heritable epimutations, including re-methylated and de-methylated, were erased and subsequently reestablished, but not unaltered, during offspring embryonic reprogramming. However, their reestablishment proportions and levels in the PS stage were altered. The heritable re-methylated epimutations had higher methylation reestablishment proportion, while the heritable de-methylated epimutations had lower methylation reestablishment proportion, when compared with the un-heritable epimutations. Meanwhile, most of the heritable de-methylated epimutations had lower reestablishment levels in stress group when compared with control group, whereas most of the heritable re-methylated epimutations had higher reestablishment levels. In addition, the DNA methylation patterns of the heritable epimutations were almost fully cleared in PGCs. Thus, it is likely that some other mechanisms participated in “marking” the heritable status of these heritable epimutations. Histone covalent modifications, such as H3K4me3 and H3K27me3, and sncRNAs, such as miRNAs and tsRNAs, can mediate inheritance of environmental-factors-induced health risks in mammals in a similar manner to DNA methylation. The roles they play in mediating psychological stress-induced paternal inheritance of health risks, through interaction with DNA methylation or marking the heritable status, require further investigation.

Long-term psychological stress likely affected not only the paternal generation but also the offspring. In this study, we provide compelling evidence that psychological stress induces the storage of “epigenetic memory” in the paternal germ cells. Although the majority of the DMRs vanished after removal of the original stimulus, significant proportions were shown to be intergenerationally and transgenerationally transmitted. These data provide strong evidence contradicting the hypothesis presented in a recent review, that the majority of environmental factor-induced or sporadically arising DMRs either were not transmissible or were only rarely transmissible^25^. Our findings reported here have shown that notable portions of these defects were paternally inherited. Epigenetic inheritance of these “epigenetic memory” produced offspring with the potential to be adapted to environmental challenges that their parents experienced, with major implications for heredity and evolution. Furthermore, these heritable “epigenetic memory” are transmitted to germ cells of the offspring, but also be inherited by other tissues that modulate the expression patterns of the relevant genes. These findings thus provide a likely mechanism underlying paternal inheritance of psychological post-stress effects (see the model in Fig. 7a). However, the downward trend of the differences in DNA methylation levels between control and stress groups indicated that inheritance of these “epigenetic memory” was reduced in advanced generations after removal of the original stimulus. Thus, consecutive environmental stimuli should be maintained for long-term adaptation and evolution of the subsequent generations.

The reprogramming process between generations represented the largest hurdle to conceptualizing epigenetic inheritance^20^. To date, the mechanism underlying these epigenetic alterations that evaded offspring embryonic reprogramming remained unknown. Our most interesting findings were that the heritable DMRs were erased and subsequently reestablished, but not unaltered, thereby surviving reprogramming to mediate paternal inheritance of stress-induced health risks. However, their DNA methylation reestablishment proportions and levels were altered during the reprogramming process in the PS stage (Fig. 7b). This also implied that the PS stage would be the crucial period for determining epigenetic inheritance. This result was different from earlier studies suggesting that if epigenetic markers were to be maintained across generations, they were carried forward at the ICM stage, since some imprinted genes and repeated elements escaped from demethylation during this period^46,51^. Different observations were possibly due to different inheritance mechanisms between canonical genes and the imprinted and repeated elements.

Furthermore, DNA methylation patterns of these heritable DMRs were almost fully cleared in PGCs. Thus, there were likely some other mechanisms “marking” the heritable status of these DMRs. Recent studies have shown that sncRNAs such as miRNAs, tsRNAs, rsRNAs, and histone covalent modifications such as H3K4me3 and H3K27me3 can mediate the inheritance of environmental factor-induced phenotypic changes in mammals in a similar manner to that of the more widely studied DNA methylation^5,7,29–32^. Our results supported the hypothesis that stress-induced differentially enriched sncRNAs in paternal sperm might play an important role in the occurrence and paternal inheritance of stress-induced DMRs. However, their interactions in mediating environmental factors (such as psychological stress)-induced paternal inheritance of health risks) need further investigation (Fig. 7b). The situation is the same as in histone covalent modifications that have been reported to enable maintenance of paternal DNA methylation and reprogramming of maternal DNA methylation in zebrafish embryos^52^.

In summary, long-term psychological stress (“paternal life experiences”) not only induced health risks (disorders of metabolism, behavior, and reproduction) on the parents themselves but also stored a good deal of environmental information (“epigenetic memories”) in their germ cells. These “epigenetic memories” survived offspring embryonic reprogramming through erasure and subsequent reestablishment, but not through unaltered, to mediate germline inheritance of “epigenetic memories”. However, their reprogramming patterns were altered in the PS stage. Furthermore, there was tissue inheritance of this “epigenetic memories” that mediated paternal inheritance of stress-induced health risks. Taken together, our results thus support the hypothesis that reprogramming-altered DMRs mediated paternal inheritance of long-term psychological stress-induced health risks are heritable across generations via the germline.

## Materials and methods

### Animals and chronic restraint stress procedure

This study was approved by the Institutional Animal Care Committee of Shanghai Institute of Biochemistry and Cell Biology. Male C57BL/6J-*Pou5f1*^*GFP/GFP*^ mice and female DBA/2J mice used in our studies were housed at 22 ± 2 °C with humidity of 55 ± 10% and under a 12-h light/dark cycle. In addition, the mice were allowed access to chow and water ad libitum except during the process of chronic restraint stress for the F_0_ generation treatment group.

A mouse model of chronic restraint stress was established as previously described by Uchida et al.^53^. In brief, 3-week-old male C57BL/6J-*Pou5f1*^*GFP*^ mice were randomly assigned to the control and stress groups (*n* = 6 for each group). Mice in the control group were allowed to contact each other, while those in the stress group were individually subjected to chronic-restraint stress for 2 h/day (from 11:00 AM to 1:00 PM) for 90 consecutive days in 50 mL conical centrifuge tubes with multiple punctures for air flow. During the restraint stress, mice were placed in separate sound- and light-attenuating boxes, and then immediately returned to their home cages. After chronic restraint stress treatment, those in both the control and stress groups were mated with 8-week-old DBA/2J female mice to obtain F_1_ generations. Similarly, 8-week-old F_1_ male mice were mated with 8-week-old DBA/2J female mice to obtain F_2_ generations. Only male offspring were used for subsequent studies, including DMR detection and phenotype identification to investigate paternal inheritance.

### Measurement of body weight and blood glucose level

Body weight measurement was performed weekly with a calibrated integrating scale. Fed blood glucose levels were measured using a portable blood glucose meter. To reduce the influences of stress on blood glucose levels during blood sampling and to improve data accuracy, we measured caudal vein blood glucose three times for each mouse and calculated the mean value. For the F_0_ generation, blood glucose level was detected before and after the treatment process, while for the F_1_ and F_2_ generations, blood glucose level was assayed at 4, 6, and 8 weeks.

### Behavioral assay

The behavioral assays were performed blindly without knowledge of the treatment histories of the mice. To avoid the effects of shipping stress, a 2-week habituation period was set before initiation of the behavioral testing. Meanwhile, the intervals between two tests were set as at least two days. After testing, all devices were cleaned with 70% ethanol to prevent bias caused by olfactory cues. Mice underwent the following tests: an open field test and an elevated plus maze test according to the following protocols. In the open field test, mice were positioned individually in the center of an arena (40 cm × 40 cm × 40.5 cm) in a room with dim lighting for 10 min. A video camera positioned directly above the arena was used to track the movement of each mouse. Data containing retention time in the central part and moving distance were collected. In the elevated plus maze test, the elevated plus maze task was used to assess anxiety-like behaviors in the rodents. Prior to the 10-min experiment, mice were placed in the center of the maze, and their behavior was monitored by an overhead video camera. The data were quantified to determine time spent in the central zone, open arms, and closed arms as well as the movement distance.

### Collection of oocytes and early embryos

Briefly, DBA/2J female mice aged 4–6 weeks were superovulated by intraperitoneal injection of pregnant mare serum gonadotrophin (PMSG, 5 IU), to be followed by human chorionic gonadotrophin (hCG, 5 IU) after 48 h. Oocytes at the stage of metaphase II were isolated from the ampulla of the oviduct at 12 h following hCG injection and collected in M2 medium contained hyaluronidase (Merck Millipore, Germany) to remove contaminated cumulus cells. Oocytes were then diluted in KSOM medium (Merck Millipore, Burlington, MA, USA) eight times to eliminate somatic cell pollution as described^26^. Thereafter, a microscope was used to ensure that the oocytes were uncontaminated by somatic cells. In addition, superovulated female mice were mated with male mice of the F_0_ and F_1_ generations in both the control and stress groups for collecting respective embryos of the F_1_ and F_2_ generations. The presence of vaginal plugs was considered as successful mating, and the day of observation of a plug was set as embryonic day (E) 0.5. In the current study, we collected embryos at three different stages, including inner cell mass (ICMs, E3.5), primitive streak (PS, E7.5), and primordial germ cells (PGCs, E13.5). ICMs were isolated from E3.5 blastocysts by flushing the uteri of pregnant mice with M2 medium (Merck Millipore) as described previously^26^. Then, embryos were treated with rabbit anti-mouse serum (Sigma-Aldrich, St. Louis, MO, USA) for 30 min in a CO_2_ incubator at 37 °C. Embryos were washed in KSOM and then added to standard guinea pig complement (Cedarlane) in KSOM for 30 min in a CO_2_ incubator at 37 °C. Finally, embryos were treated with acid Tyrode’s solution (Sigma-Aldrich) to remove the zona pellucida, and dead trophectoderm cells were removed from ICMs by pipetting through a fine pulled glass needle. Isolated ICMs were serially washed to remove contaminants. E7.5 embryos were isolated after mechanical dissection of the decidua from the uterine linings of mated mice. Samples were again progressively washed, and peripheral trophectodermal tissues were dissected using fine glass capillaries. PGCs were isolated from time-mated female mice carrying the *Pouf1*^GFP/GFP^ transgene expressed in the developing gonad on a C57BL/6J background. Male and female samples were collected separately, as gonads could be readily distinguished morphologically from E13.5 embryos with the same sex. PGCs were purified by a FACS Aria cell sorter with purity of >98%.

### Assessment of sperm concentration and motility

A computer-assisted sperm analysis (CASA) was employed to examine the semen quality of the male parents and their male descendants. The cauda epididymis of mice was separated from sacrificed animals, gently cut, and incubated in 500 µl M2 media in an CO_2_ incubator at 37 °C for 30 min to liberate the sperm. Then, 450 µl of the supernatant was collected and pelleted (with centrifugation at 500 g for 5 min). The sediment was thrice washed in 500 µl PBS solution to remove seminal plasma contamination. Finally, the sperm were resuspended in 200 µl M2 media, and their concentration and motility were assessed using the CASA system (Hamilton Thorne, Danvers, MA, USA) according to the manufacturer’s protocol. At least 10 imaged areas were used for data analysis for each sperm sample.

### Extraction of DNA and RNA from sperm and tissues

We randomly selected three mice from each generation of both the control group and stress group as three biological replicates. After semen quality assessment, their sperm were pelleted down by centrifugation at 500 g for 5 min and then incubated in somatic cell lysis buffer for 30 min on ice to eliminate contaminated somatic cells^5^. Sperm samples were used only if their purity reached >99.5% and were evaluated by microscopy. Sperm DNA and small RNAs were simultaneously extracted using the All Prep DNA/RNA/miRNA Universal Kit (Qiagen, Hilden, Germany) according to the manufacturer’s instructions. It was noteworthy that the dithiothreitol (DTT, 40 mM) was used to disturb the disulfide bonds of the sperm during the nucleic acid extraction process. In addition, we also dissected tissues, including testis, hippocampus, and liver to obtain DNA and RNAs. Genomic DNA and RNAs of these three tissues were extracted using an *EasyPure*^®^ Genomic DNA Kit (TransGen Biotech Co., Ltd., Beijing, China) and RNeasy Plus Universal Kits (Qiagen) according to the manufacturers’ instructions. The quality and concentration of DNA and total RNA were examined by a NanoDrop2000 (Thermo Fisher Scientific, Waltham, MA, USA).

### Resequencing of parental genomes

We performed resequencing for both male C57BL/6J-*Pou5f1*^GFP^ mice and female DBA/2J mice to identify single-nucleotide polymorphisms (SNPs) between parent genomes to determine the source of the differential DNA methylation patterns in the offspring genome from the paternal or maternal parent. Sequencing libraries were constructed using the standard Illumina protocol. Quantity and quality control of the libraries were carried out with a Qubit dsDNA HS Assay kit (Thermo Fisher Scientific) and an Agilent 2100 Bioanalyzer System (Agilent Technologies Inc, Santa Clara, CA, USA), respectively. High-quality DNA libraries were sequenced with the Illumina HiSeq X-ten. The paired-end reads had an average insert size of 300 bp. Raw reads were filtered using the clean_adapter and clean_lowqual procedures of the software fastp^54^. Then, the Burrows–Wheeler aligner (BWA v0.7.12)^55^ was used to map the clean reads to the reference Genome. SAMtools (v1.2)^56^ was employed to sort reads, and PCR duplicate reads were removed using Picard tools (v1.13, http://broadinstitute.github.io/picard/). Reads mapped to two or more places were filtered out. HaplotypeCaller was used to call SNPs and indels simultaneously via local de-novo assembly of haplotypes in an active region^57^.

### Single-cell whole-genome bisulfite sequencing

Due to the limited amount of DNA obtained from embryos and maternal oocytes for construction of bisulfite sequencing libraries, we used the scWGBS method to perform the bisulfite sequencing for one oocyte and 12 embryo samples. Briefly, 2× lysis buffer and 0.5 μl proteinase K were added to gathered cells followed by incubation at 37 °C for 1 h. Bisulfite conversion was performed on cell lysates using an EZ DNA Methylation-Gold kit as well as WGBS. After purification, DNA was eluted in 10 mM Tris-Cl and combined with oligo 1. Before incubation at 65 °C for 3 min, 50 U of Klenow exo– (Sigma-Aldrich) were added, and the samples were incubated at 4 °C for 5 min and at 37 °C for 30 min. Samples were incubated at 95 °C for 1 min and transferred immediately to ice before the addition of fresh oligo 1 (10 pmol), Klenow exo– (25 U), and dNTPs (1 nmol) in a 2.5 μl total. The samples were incubated at 4 °C for 5 min and at 37 °C for 30 min. The random priming and extension were repeated a further three times (five rounds in total). Samples were then incubated with 40 U exonuclease I (New England Biolabs) for 1 h at 37 °C before DNA was purified using 0.8× Agencourt Ampure XP beads (Beckman Coulter, Brea, CA, USA). Samples were eluted in 10 mM Tris-HCl (pH 8.5) and incubated with washed Streptavidin Dynabeads M-280 (Life Technologies, Carlsbad, CA, USA) for 20 min with rotation at room temperature. Beads were twice washed and resuspended in dNTPs, 0.4 μM oligo 2 and 1× Blue Buffer. Samples were incubated at 95 °C for 45 s and transferred immediately to ice before addition of 100 U Klenow exo– and incubation at 4 °C for 5 min, +1 °C/15 s to 37 °C, 37 °C for 90 min. Libraries were then PCR-amplified as follows: at 95 °C for 2 min, 12–13 cycles of (at 94 °C for 80 s, at 65 °C for 30 s, at 72 °C for 30 s), at 72 °C for 3 min, and a 4 °C hold. Purified libraries were assessed for quality and quantity using Agilent Bioanalyzer and StepOnePlus Real-Time PCR System. Single cell libraries were prepared for 125-bp paired-end sequencing on a HiSeq 2500.

### Bisulfite sequence data filtering, alignment, and quantification of methylation levels

The raw data derived from both WGBS and scWGBS were filtered to obtain clean reads. Adapter-polluted reads, low-quality reads, and reads with over 10% Ns were removed. Clean reads were mapped to the reference genome (mm10, UCSC) by Bismark (ver. 2.2.3)^58^, and only uniquely mapped reads were retained. Cytosines were considered to be methylated based on the binomial test followed by adjusting the false discovery rate using the Benjamini–Hochberg method. The methylation level of a single cytosine was calculated as mC/(mC+umC), where mC was the number of methylated reads and umC was the number of the unmethylated reads.

### Identification of differentially methylated regions

DMRs were identified using Bioconductor package DSS (v2.14.0) which uses biological replicates and information from CpG sites across the genome to stabilize the estimation of the dispersion parameters^59^. The core of DSS is a procedure based on Bayesian hierarchical model to estimate and shrink CpG site-specific dispersions, then conduct Wald tests for detecting differential methylation. Only CpGs that were covered at least fivefold were considered for a given comparison. We first performed statistical tests of differentially methylated loci using *DMLtest* function (smoothing = TRUE, smoothing.span = 500) in DSS; the results were then used to detect differentially methylated cytosines (DMCs) with the parameters: absolute delta beta value (|Δβ|_DMC_) > 0.1, p.threshold <0.01 (the threshold for *p*-value here actually refers to local false discovery rate-FDR). The DMCs were then merged into blocks if they both showed similar methylation patterns using *callDMR* function with the parameters: |Δβ|_DMR_ > 0.1, the minimum length for a DMR ≥ 50 bp, the minimum number of CpG sites for DMR ≥ 4 and p.threshold (FDR) < 0.01. Merging nearby CpG sites (refer in particular to not differentially methylated CpG sites) into blocks can cause some regions with methylation difference smaller than theoretical value. Thus, we further filtered out DMRs with standards: |Δβ|_DMR_ ≥ 0.1 or variated >20% (as some of the regions changed from unmethylated/low-methylated to methylated/high-methylated. Although the |Δβ|value was small, their alterations were relatively large when compared to their original status). DMRs-related genes were then annotated using bedtools^60^. We set the overlap length to at least one base when investigate the enriched region of DMRs on genome elements. The enrichment analysis in GO terms (http://geneontology.org/) or KEGG pathways (http://www.kegg.jp/) was based on a hypergeometric test with threshold *q* < 0.05 by the clusterProfile package in R^61^ to identify significantly enriched genes.

### Identification of heritable DMRs

Two statistical approaches were used to analyze inherited DMRs. Wilcoxon signed-rank tests were performed based on mean DNA methylation levels of candidate regions in all three generations. In brief, the DNA methylation levels of the regions that were differentially methylated in the F_0_ generation (i.e., F_0_-DMRs) were calculated in all three generations based on cytosine residues with sequencing coverage ≥5 in the regions. Then, three generations were considered to be three biological replicates, and significant differences in DNA methylation levels were analyzed by single-tailed paired-comparison tests. A region with FDR < 0.01 and |Δβ|value ≥0.1 or variated >20% was considered a transgenerational inherited DMR. The same analysis was undertaken on intergenerationally inherited DMRs on the basis of mean DNA methylation levels of candidate regions in both F_0_ and F_1_ generations. The other analysis involved unpaired *t*-tests based on mean DNA methylation levels of three replicates in each generation. Briefly, significant differences in DNA methylation levels of each generation were analyzed by Student’s *t*-test on the basis of three biological replicates. A region with FDR < 0.01 and |Δβ|values ≥0.1 or variated more than 20% in all three generations was considered as a transgenerationally inherited DMR. The same analysis was conducted on intergenerationally inherited DMRs.

### Collection of phenotype-associated genes, imprinted genes, and transposable elements

Phenotype-associated genes in mice were collected from the Mouse Genome Informatics database (http://www.informatics.jax.org/)^62^ using the following keywords: glucose metabolism, reproduction, body weight, and emotional and social behavior. The mouse imprinted genes were obtained from the geneimprint database (www.geneimprint.com/site/genes-by-species.Mus+musculus). The CpG islands in these imprinted genes were annotated by the UCSC database (http://genome.ucsc.edu/index.html). Transposable elements were gathered from the Dfam database (https://www.dfam.org/home)^63^, including long-terminal repeated (LTR) retrotransposons, long interspersed nuclear element (LINE) retrotransposons, short interspersed nuclear element (SINE) retrotransposons, satellite repeats, pseudogenes, and DNA transposons. We merged the intersecting repeats into a long repeat if they belonged to the same type.

### sncRNA sequencing and data processing

Small RNAs extracted from sperm samples were used to prepare a sequencing library using NEBNext Small RNA Library Prep kit for Illumina (New England Biolabs) according to the manufacturer’s instructions. Amplified libraries were cleaned using Agencourt AMPure XP (Beckman Coulter), and RNAs with corresponding size were selected on pre-cast 6% polyacrylamide Novex TBE gels (Invitrogen, Carlsbad, CA, USA). Disintegrated gels were incubated at 37 °C for 1 h on a shaker and were quickly frozen for 15 min at −80 °C, followed by another incubation for 1 h. The libraries were then precipitated overnight at −80 °C by adding 1 μl of GlycoBlue (Invitrogen), at 0.1 times the volume of acetate 3 M (pH 5.5) and three times the volume of 100% ethanol. After determining of library concentration using the QuantiFluor ONE dsDNA system on a Quantus fluorometer (Promega, Madison, WI, USA), the libraries were sequenced on an Illumina NextSeq 500 platform with a NextSeq 500/550 High Output Kit v2 (75 cycles) (Illumina).

Raw data were filtered sequentially as follows: removal of the reads without a 3′adapter; removal of the reads without insert fragments; removal of the reads with excess A/T; removal of the reads with a length out of range; and removal of the low-quality reads. The data were processed using a fastp script^54^ with default parameters to cut adapters and filter low-quality reads. Only trimmed reads between 15 and 50 nucleotides and with 80% of the nucleotides showing Illumina quality scores (Q-scores) >20 were retained as clean reads.

Afterwards, the clean reads were aligned to the reference genome (mm10), a microRNA (miRNA) database (http://www.mirbase.org/index.shtml), a ribosomal RNA (rRNA) database (https://www.ncbi.nlm.nih.gov/nuccore), a genome transfer RNA (GtRNA) database (http://gtrnadb.ucsc.edu/), a mitochondrial tRNA (mitotRNA) database (http://mttrna.bioinf.uni-leipzig.de/mtDataOutput/), the PIWI-interacting RNA (piRNA) database (http://www.regulatoryrna.org/database/piRNA/), the ensemble non-coding (ncRNA) database (http://www.ensembl.org/index.html), and the Rfam database (http://rfam.xfam.org/) to validate the source of the small non-coding RNAs (sncRNAs) using Sports 1.1 (https://github.com/junchaoshi/sports1.1)^36,64^. Differentially expressed miRNAs and tsRNAs were analyzed based on subclasses (Table S3) and normalized to RPM. Student’s *t*-test was used for making comparisons, and *P* < 0.05 was considered statistically significant. Statistics were based on the following conditions: the sum of the reads count >15 and the sum of the RPM values >1 for all six samples.

Differentially expressed sncRNA sequences, including rsRNAs, miRNAs, tsRNAs, were extracted from sequencing data. Screening was performed according to the following conditions: sum of the reads count >15; sum of the RPM value >1 for all six samples, and *P* < 0.05 between the control and the stress groups. Sequences were subsequently aligned to stress-induced F_0_-DMRs sequences via direct matching and base-complementation pairing using a 7-bp seed window. The differentially expressed sncRNA sequences that were aligned to DMRs were counted. Meanwhile, the longest alignment was recorded as the matching length.

### Target predication of differentially expressed miRNAs and tsRNAs

Target predication of differentially expressed miRNAs was conducted using the Targetscan database (http://www.targetscan.org/mmu_72/)^65^, predicting targets of conserved miRNA families, including positions on UTRs (without gaps), and UTR multi sequence alignments (MSA; with gaps).

In *Drosophila*, tsRNAs modulate the expression of genes via conserved antisense sequence matching in an additive manner occurring primarily at the translational level^66^. We thus downloaded the mouse mRNA sequences from the National Center for Biotechnology Information (NCBI) database (http://hgdownload.cse.ucsc.edu/goldenPath/mm10/bigZips/mrna.fa.gz), and aligned differentially expressed tsRNA subclasses to these mRNA sequences using the requirement of 7-mer antisense perfect match with bowtie2^67^.

### Bisulfite sequencing PCR method to validate DMRs

Some of the differential methylation regions identified using WGBS were validated by BSP. DNA methylation patterns of some DMRs associated with phenotypes were investigated in tissues using BSP method. In brief, bisulfite conversion was conducted with EpiMark Bisulfite Conversion kit (NEB) according to the manufacturer’s instructions. EpiMark Hot Start Taq (NEB) was used for subsequent PCR amplification. After purification with EasyPure Quick Gel Extraction kit (TransGen), the PCR product was cloned into a pEasy-T1 vector (TransGen) and sequenced. DNA methylation status of the targeted region was analyzed on the QUMA website (http://quma.cdb.riken.jp/). The primers used in this study were listed as follows: Rhobtb3-BSP-For, 5′-TTATTAGGTTTAGGTATTGTGTAGTTTTTAATT-3′, Rhobtb3-BSP-Rev, 5′-AATCATAAATCCTTCAACTTTATATCTTTCTAT-3′; Il12rb1-BSP-For, 5′- TTTTTTAGTTAGGGTAGGAATAGGGTATATAT-3′, Il12rb1-BSP-Rev, 5′-CACTCAAAATCAACAACATCTCTACCCACAA-3′; Ddo-BSP-For, 5′- AAAGAGAGGGGAGAGGTATGTGTTATTGAAG-3′, Ddo-BSP-Rev, 5′- CCCAAATTATAATCTACTAACCAAACACAAC-3′; Oprm1-BSP-For, 5′-GTTGATAGATTTGAAATTTAAATTTAGATT-3′, Oprm1-BSP-Rev, 5′- ACTAATTAAAAAATTACTAACACACATATA-3′.

### Identification of gene expression patterns using qPCR

We used qPCR to identify the gene expression patterns related to heritable DMRs in phenotype-associated tissues. For this purpose, 1 μg total RNA was used to synthesize cDNA with an EasyScript One-Step gDNA Removal and cDNA Synthesis SuperMix kit (TransGen) according to the manufacturers’ instructions. The PCR was performed using *TransStart*^®^ Top Green qPCR SuperMix (TransGen). The qPCR volume contained 0.5 μl of synthesized cDNA, 10 μl of 2x TransStart Top Green qPCR SuperMix (TransGen), 1.6 μl of forward primer (2.5 μM), 1.6 μl of reverse primer (2.5 μM), 0.4 μl of Passive Reference Dye II (50 x), and ddH_2_O to a final volume of 20 μl. The PCR procedure included an initial step of 95 °C for 30 s; 40 cycles of 95 °C for 5 s, and 60 °C for 34 s; and a dissociation curve step on an ABI Prism 7500 Real-Time Thermal Cycler (Applied Biosystems, Foster city, CA, USA). The results were normalized to *Gapdh*. The primers used in this study were listed as follows: Rhobtb3- RT-qPCR -For, 5′-TTGGAGGAGTGCTGGAGTA-3′, Rhobtb3- RT-qPCR -Rev, 5′-CGGAGTGATAGTGTGATGCC-3′; Il12rb1- RT-qPCR -For, 5′-TACAAGGTTCAGGTGCGA-3′, Il12rb1- RT-qPCR -Rev, 5′-ATGTATCCGAGACTGCCCA-3′; Ddo- RT-qPCR -For, 5′-ACAACCCTGAAGTGCGAGAC-3′, Ddo- RT-qPCR -Rev, 5′-TCCTGGTGAGTAGCAGACCTC-3′; Oprm1- RT-qPCR -For, 5′-CTTGTAAGAAACTGACGGAGC-3′, Oprm1- RT-qPCR -Rev, 5′-TGGTTCTGAATGCTTGCTG-3′; mGapdh- RT-qPCR -For, 5′-AAATGGTGAAGGTCGGTG-3′, and mGapdh- RT-qPCR -Rev, 5′-ATTTGCCGTGAGTGGAGT-3′.

## Supplementary information

Supplemental materials

Supplemental Table S1

Supplemental Table S2

Supplemental Table S3

Supplemental Table S4

## Data Availability

All datasets generated in this work have been deposited in the Sequence Read Archive (SRA) database under the accession number PRJNA662676, Gene Expression Omnibus (GEO) accession number GSE185579.
