# Supplementary material for: Sperm epigenetic alterations contribute to inter- and transgenerational effects of paternal exposure to long-term psychological stress via evading offspring embryonic reprogramming
Source: Cell Discov. 2021 Oct 27;7:101. doi: 10.1038/s41421-021-00343-5 (PMC8553786; doi:10.1038/s41421-021-00343-5)
Supplement: Supplementary file 1 — Supplemental materials [file 41421_2021_343_MOESM1_ESM.pdf]

Supplementary information for

Sperm epigenetic alterations contribute to inter- and transgenerational effects of paternal exposure to long-term psychological stress via evading offspring embryonic reprogramming

Xiaoguo Zheng[1,2,†], Zhenhua Li[1,2,†], Guishuan Wang[3,†], Hanshu Wang,[1,2], Yuchuan Zhou[1,2], Xinzhi Zhao[1,2], C. Yan Cheng[4,*], Yunbo Qiao[5,*], Fei Sun[1,2,3,6,*]

1. International Peace Maternity & Child Health Hospital, School of Medicine, Shanghai Jiao Tong University, Shanghai, 200030, China.

2. Shanghai Key Laboratory of Embryo Original Disease, Shanghai, China.

3. Medical School, Institute of Reproductive Medicine, Nantong University, Nantong, Jiangsu, China.

4. The Mary M. Wohlford Laboratory for Male Contraceptive Research, Center for Biomedical Research, Population Council, 1230 York Ave, New York 10065, USA

5. Precise Genome Engineering Center, School of Life Sciences, Guangzhou University, Guangzhou, 510006, China.

6. Key Laboratory of Fertility Preservation and Maintenance of Ministry of Education, School of Basic Medicine, Ningxia Medical University, Yinchuan, 750004, China

[†]These three authors contributed equally

*Corresponding author: Fei Sun, E-mail: sunfei@ntu.edu.cn

Yunbo Qiao, E-mail: ybqiao84@163.com

C. Yan Cheng, E-mail: ccheng@mail.rockefeller.edu

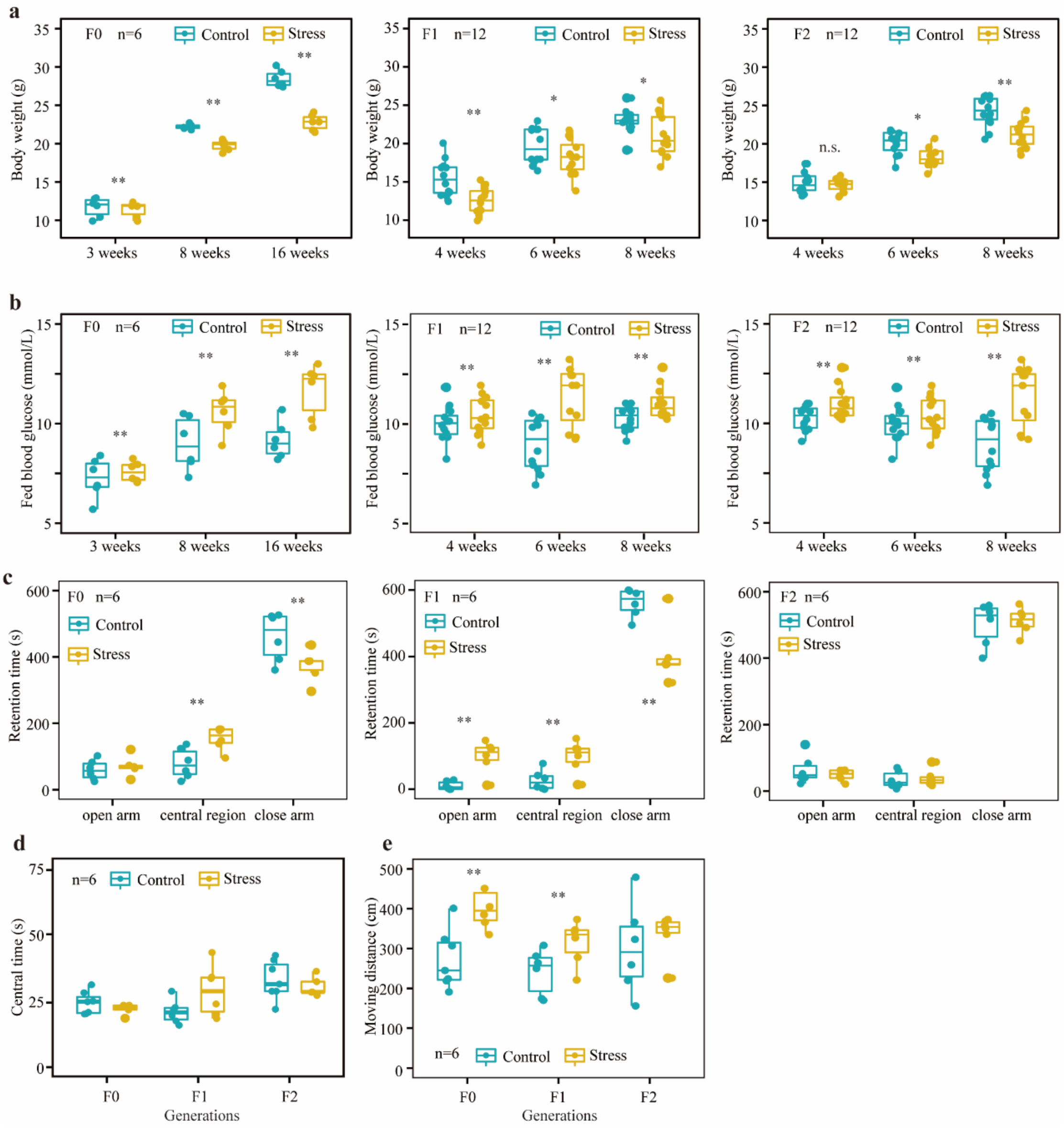

Supplementary Fig. S1. Chronic psychological stress induced paternal inheritance of developmental, metabolic, behavioral, and reproductive disorders in mice. a, Stress-induced transgenerational inheritance of developmental retardation. **b**, Stress-induced transgenerational inheritance of disorder in blood glucose metabolism. **c**, Stress-induced intergenerationally inherited behavioral disorder. Elevated plus maze (EPM) test to assess anxiety-like behavior of the mice. **d**, Open field test to assess anxiety-like behavior of the mouse. **e**, Difference in moving distances between stress and control groups during the open field test. **: *t*-test *p*-value <0.01; n.s.: no significant, *t*-test *p*-value >0.05.

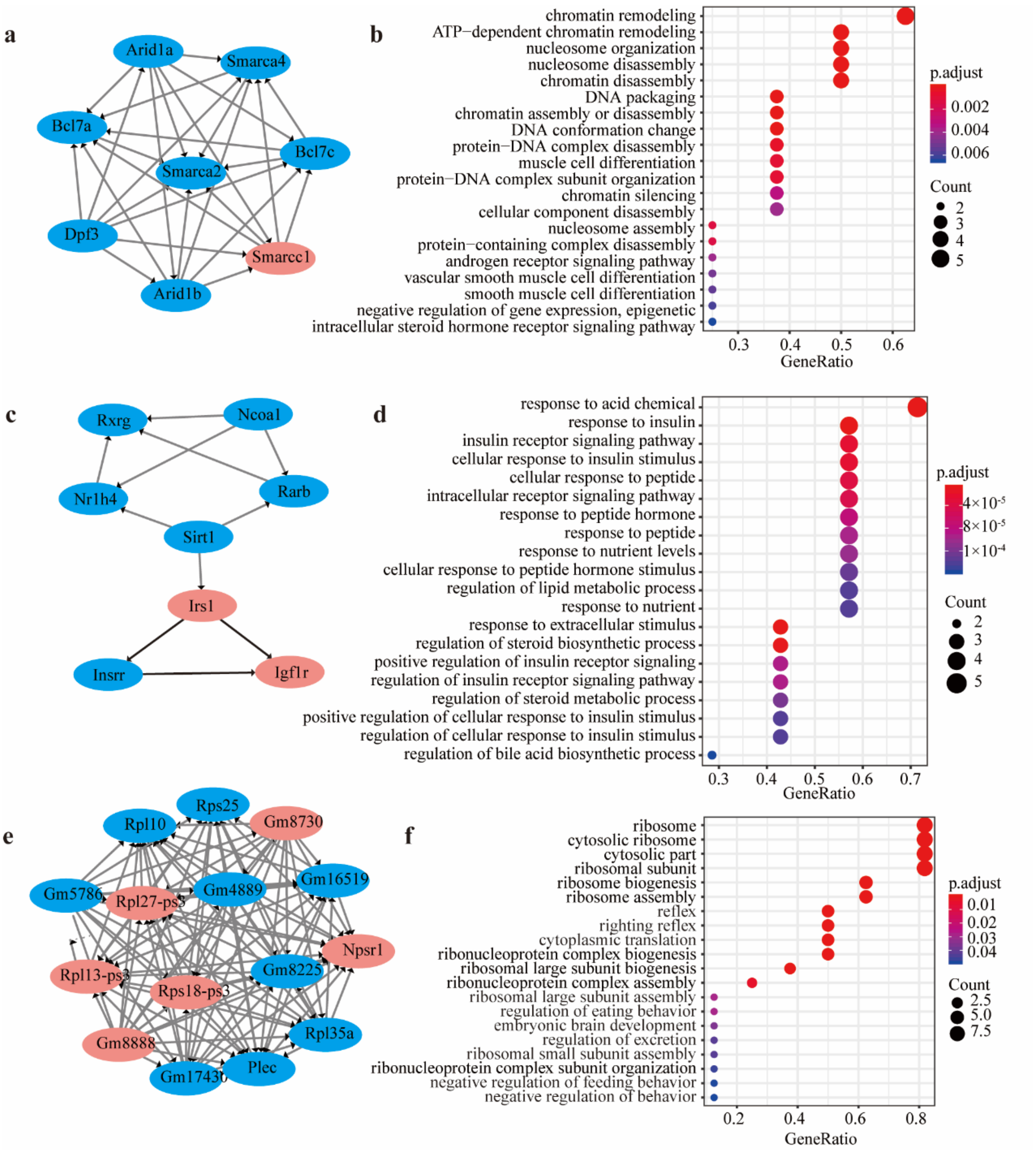

Supplementary Fig. S2. Stress-induced DMRs associate with paternally inherited phenotypes. **a**, Interaction of the genes that enriched in MCODE subgroup 1. Red nodes represented genes that related to re-methylated DMRs, while blue nodes represented genes that related to de-methylated DMRs. **b**, Gene Ontology (GO) analysis of the genes in subgroup 1. **c**, Interaction of the genes that enriched in subgroup 2. **d**, GO analysis of the genes in the subgroup 2. **e**, Interaction of the genes that enriched in subgroup 3. **f**, GO analysis of the genes in the subgroup 3.

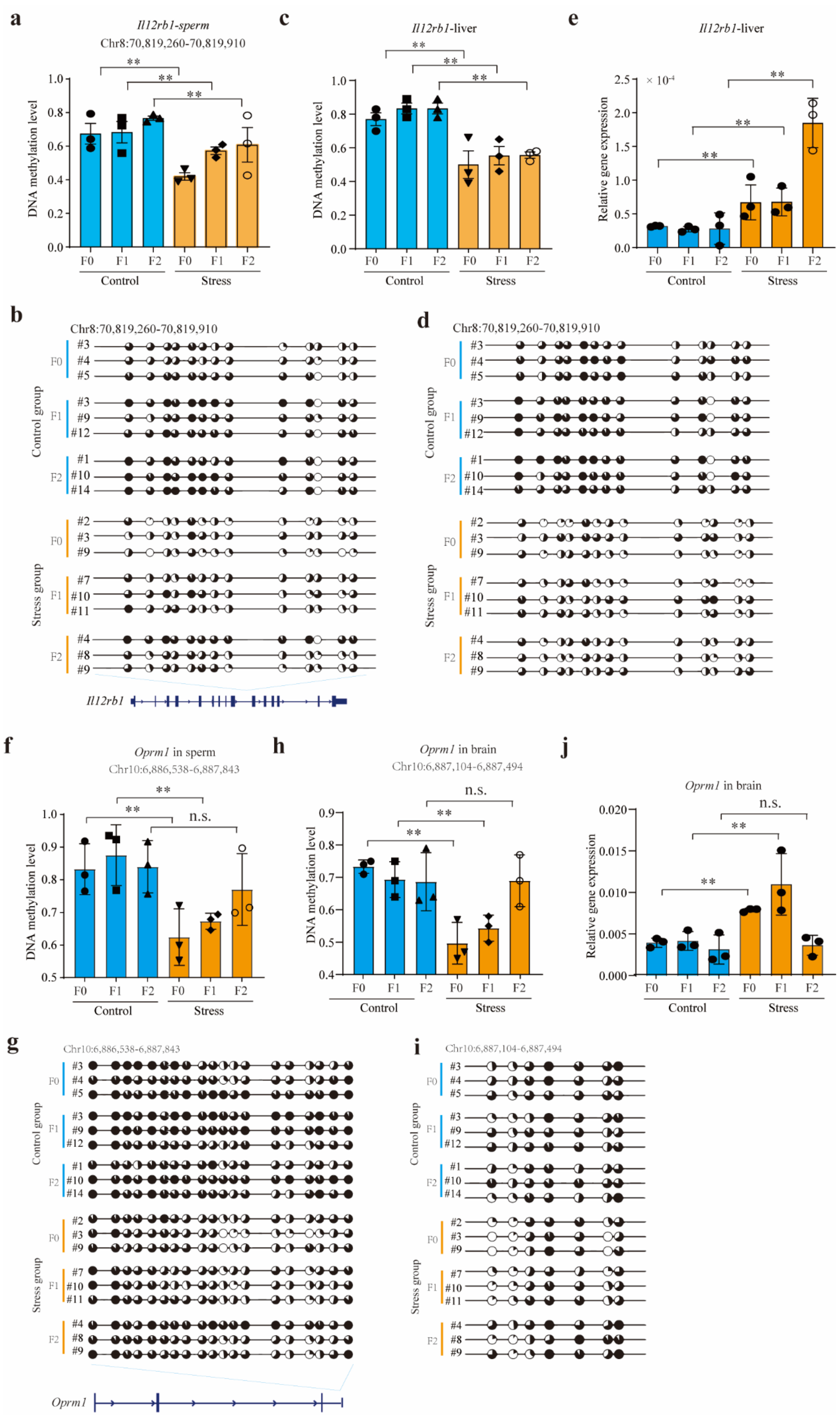
a
Il12rb1-sperm
Chr8:70,819,260-70,819,910
DNA methylation level
Control
Stress
b
Chr8:70,819,260-70,819,910
Control group
Stress group
Il12rb1
c
Il12rb1-liver
d
Chr8:70,819,260-70,819,910
e
Il12rb1-liver
Relative gene expression
f
Oprm1 in sperm
Chr10:6,886,538-6,887,843
n.s.
g
Chr10:6,886,538-6,887,843
Oprm1
h
Oprm1 in brain
Chr10:6,887,104-6,887,494
i
Chr10:6,887,104-6,887,494
j
Oprm1 in brain

Supplementary Fig. S3. Epigenetically inherited DMRs not only were transmitted to sperm, but also were inherited by tissues, and subsequently mediated expression alterations of their related genes. DNA methylation levels **(a)** and patterns **(b)** of a transgenerationally-inherited de-methylated DMR, Chr8:70,818,260-70,819,910, in sperm samples of all generations. DNA methylation levels **(c)** and patterns **(d)** of this DMR in liver tissues. **e**, Expression patterns of the *Il12rb1* in liver tissues. DNA methylation levels **(f)** and patterns **(g)** of an intergenerationally-inherited de-methylated DMR, Chr10:6,886,538-6,887,843, in sperm samples of all generations. DNA methylation levels **(h)** and patterns **(i)** of this DMR in brain tissues. **j**, Expression patterns of the *Oprm1* in brain tissues. **: *t*-test p-value <0.01; n.s.: no significant, *t*-test *p*-value >0.05.

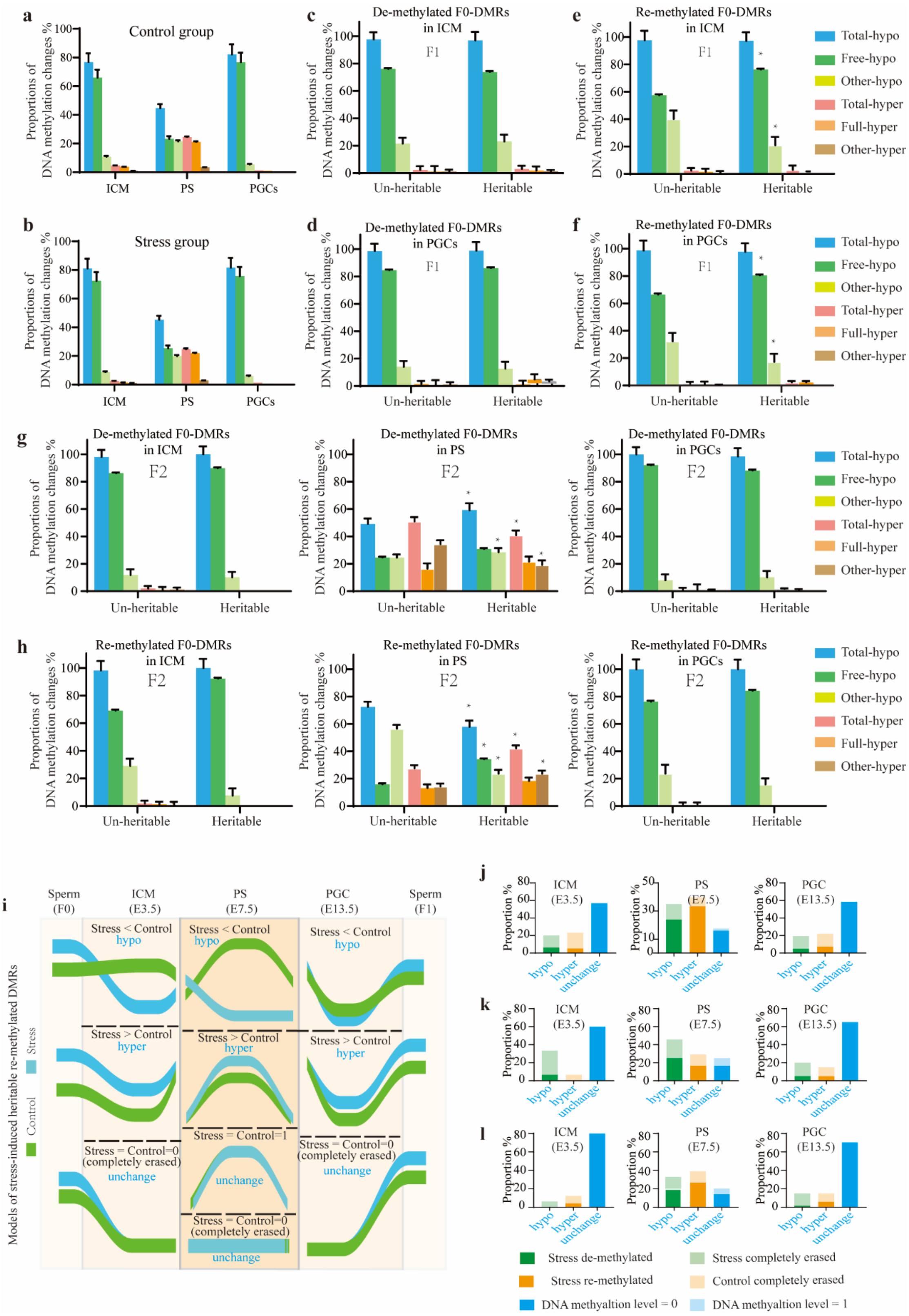

Supplementary Fig. S4. DNA methylation reestablishment proportions and levels of the heritable DMRs have been altered in the PS stage. DNA methylation alterations in embryo samples in in comparative analysis with the paternal sperm in control group **(a)** and stress group **(b)**. Dynamic DNA methylation patterns of both heritable and un-heritable de-methylated F0-DMRs in F1-ICM **(c)** and F1-PGC **(d)**. Dynamic DNA methylation patterns of both heritable and un-heritable re-methylated F0-DMRs

in F1-ICM **(e)**, F1-PGC **(f)**. Dynamic DNA methylation patterns of both heritable and un-heritable de-methylated F0-DMRs **(g)** and re-methylated F0-DMRs **(h)** in all three embryonic stages of the F2 generation. **i**, Dynamic DNA methylation models of the stress-induced heritable re-methylated DMRs during reprogramming process. In each stage, all DMRs were classified into three types: hypo (stress group was de-methylated), hyper (stress group was re-methylated), and unchanged (there was no difference between two groups). **j**, Proportion of each DMR type in each embryo stage of the heritable re-methylated DMRs in the F1 generation. **k**, Proportion of each DMR type in each embryo stage of the heritable de-methylated DMRs in the F2 generation. **l**, Proportion of each DMR type in each embryo stage of the heritable re-methylated DMRs in the F2 generation.

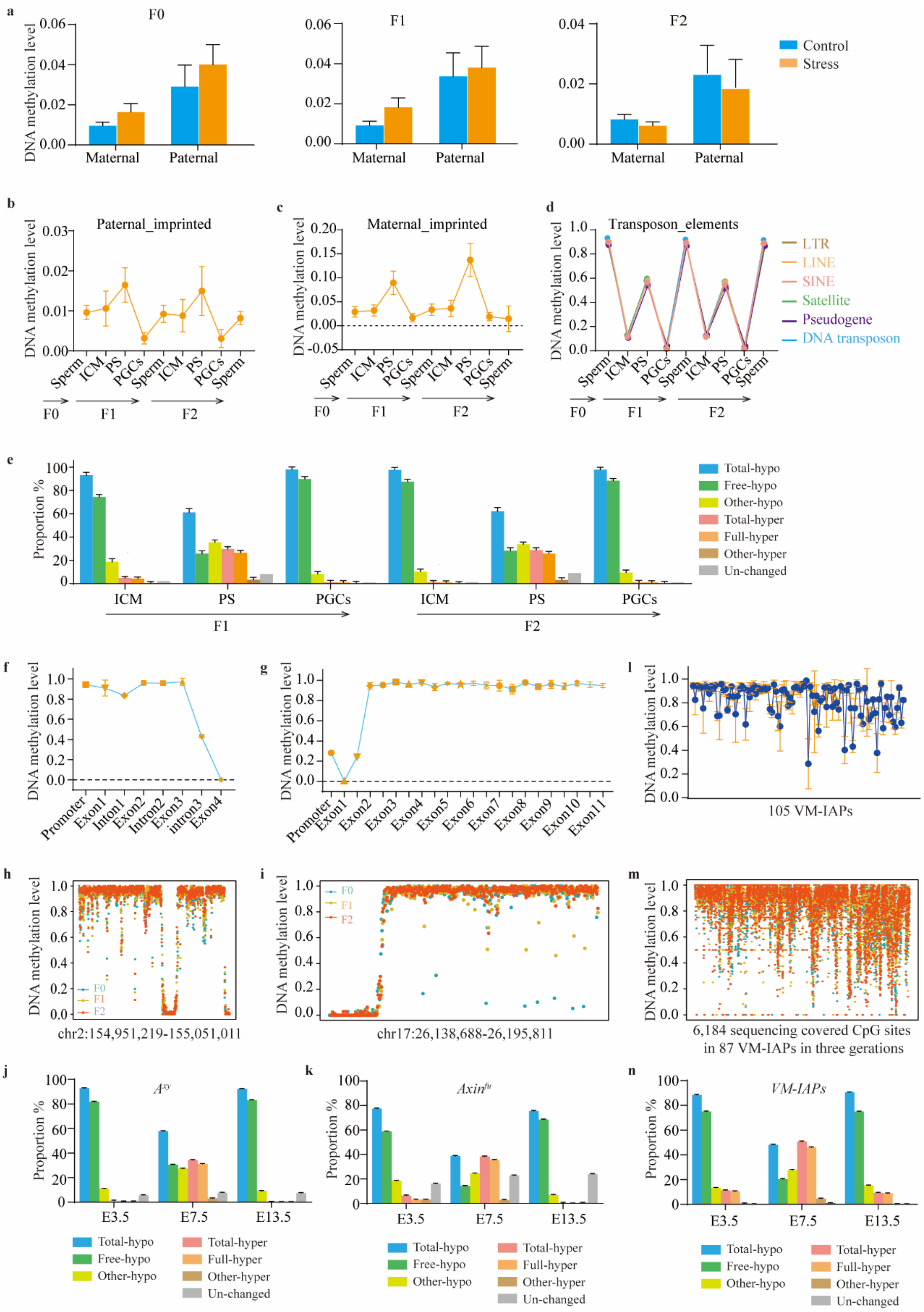

Supplementary Fig. S5. Dynamic DNA methylation patterns of the imprinted genes and transposable elements during reprogramming. a, DNA methylation levels of both paternal and maternal imprinted genes in all three generations. DNA methylation patterns of the paternal imprinted genes **(b)**, maternal imprinted genes **(c)** and transposable elements **(d)** in sperm and embryo samples of both F_1 and F_2 generations. **e,** Dynamic DNA methylation patterns of the transposable elements during reprogramming. DNA methylation patterns of the *Agouti* locus **(f)** and the *Fused* locus

(g). Distribution of the DNA methylation levels of all CpG sites in the *Agouti* locus **(h)** and the *Fused* locus **(i)** in all three generations. Dynamic DNA methylation patterns of the *Agouti* locus **(j)** and the *Fused* locus **(k)** during reprogramming. **l,** Distribution of the DNA methylation levels of the 105 variably methylated IAPs (VM-IAPs). **m,** Distribution of the DNA methylation levels of all CpG sites in these VM-IAPs. **n,** Dynamic DNA methylation patterns of these VM-IAPs during reprogramming.
